# Elevated level of mitochondrial reactive oxygen species via fatty acid β-oxidation in cancer stem cells promotes cancer metastasis by inducing epithelial–mesenchymal transition

**DOI:** 10.1186/s13287-019-1265-2

**Published:** 2019-06-13

**Authors:** Caihua Wang, Liming Shao, Chi Pan, Jun Ye, Zonghui Ding, Jia Wu, Qin Du, Yuezhong Ren, Chunpeng Zhu

**Affiliations:** 1 Department of Gastroenterology, The Second Affiliated Hospital, ZhejiangUniversity School of Medicine, Hangzhou, 310009 China; 2 Department of Surgical Oncology, The Second Affiliated Hospital, Zhejiang University School of Medicine, Hangzhou, 310009 China; 3 Department of Biochemistry and Molecular Biology, Mayo Clinic, Arizona, AZ 85259 USA; 4 Department of Endocrinology and Metabolism, The Second Affiliated Hospital, Zhejiang University School of Medicine, Hangzhou, 310009 China

**Keywords:** Cancer stem cells, Tumor sphere, ROS, Cancer metastasis, Mitochondria, Epithelial-mesenchymal transition

## Abstract

**Background:**

Cancer stem cells (CSCs) play a critical role in tumor development and progression and are involved in cancer metastasis. The role of reactive oxygen species (ROS) in CSCs and cancer metastasis remains controversial. The aim of the present study was to investigate the correlation between ROS level of CSCs and cancer metastasis and to explore the possible underlying molecular mechanisms.

**Methods:**

Four different cell lines were used to isolate tumor spheres and to analyze intrinsic properties of tumor sphere cells including proliferation, self-renewal potential, differentiation, drug-resistance and cancer metastasis in vitro and in vivo. ROS assays were used to detect the intracellular ROS level of tumor spheres cells. Gene expression analysis and western blot were used to investigate the underlying mechanisms of ROS in regulating cancer metastasis.

**Results:**

Tumor spheres possessed the characteristic features of CSCs, and ROS-high tumor spheres (RH-TS) displayed elevated mitochondrial ROS level exclusively drove metastasis formation. The gene expression analysis showed elevated fatty acid β-oxidation, downregulation of epithelial marker upregulation of mesenchymal markers, and the activation of MAP kinase cascades. Furthermore, 14 up-regulated genes in RH-TS cells were associated with reduced overall survival of different cancer patients.

**Conclusions:**

Our findings demonstrate that CSCs characterized by elevated mitochondrial ROS level potentiate cancer metastasis. Mechanistically, elevated mitochondrial ROS via fatty acid β-oxidation, activates the MAPK cascades, resulting in the epithelial-mesenchymal transition (EMT) process of RH-TS cells, thereby potentiating caner invasion and metastasis. Therefore, targeting mitochondrial ROS might provide a promising approach to prevent and alleviate cancer metastasis induced by RH-TS cells.

**Electronic supplementary material:**

The online version of this article (10.1186/s13287-019-1265-2) contains supplementary material, which is available to authorized users.

## Background

Although only 0.01–0.02% of potential cancer cells succeed in metastatic colonization, more than 90% of mortality from cancer is attributable to metastases [1, 2]. It is vital to define and separate these life-threatening metastatic cancer cells. As tumors progress, intra-tumor heterogeneity arises through cycles of mutation and clonal selection. Most tumors contain a subpopulation of highly tumorigenic cells, known as cancer stem cells (CSCs) or tumor-initiating cells (TICs). CSCs, an integral part of tumor mass, are generally supposed to be a sub-population of quiescent cells endowed with self-renewal and differentiation properties and strongly influence tumor propagation and cancer metastasis [3–5]. The discovery of cancer stem cells has significantly changed our concepts about the mechanisms of multi-step tumorigenesis and metastasis, since these self-renewing cells (or closely related progenitor cells), rather than the bulk populations of cancer cells, may be the objects of genetic alteration and clonal selection.

Early reports suggested that CSCs were mostly homogeneous, which were commonly characterized on the basis of the expression profiles of their markers. However, subsequent works revealed an unexpected heterogeneity within the CSCs compartments from different tumors, including breast cancer [6–8], melanoma [9], colorectal cancer [10], and glioblastoma [11]. Intra-tumor heterogeneity allows CSCs to differentiate into multiple tumor cell types and contributes to the resistance of the targeted therapy. Accordingly, strategy which specifically targets and kills metastasis-related CSCs represents one of the most important challenges for tumor eradication.

Recently, targeting cancer via reactive oxygen species (ROS)-based mechanisms has been argued as a radical therapeutic approach [12]. Cancer cells frequently display higher level of ROS than normal cells, implicating that targeting cancer via enhancing ROS might selectively kill cancer cells. However, the role of ROS in cancer metastasis has been of considerable debate. Several studies concluded that ROS, as signaling molecules, facilitated CSCs self-renewal and expansion [13, 14], stimulated cell invasiveness [15], and potentiated tumor progression [16, 17]. Nonetheless, increased ROS level was also reported to be detrimental for other stem cells such as hematopoietic stem cells (HSCs) [18] and inhibit cancer metastasis [19]. The illusive roles of ROS in cancer metastasis might partially result from heterogeneity of the CSCs.

As hypothesized, CSCs might divide slowly and maintain a lower metabolic state and lower ROS level than non-CSCs [20–22]. However, it remains unknown whether all CSCs are equally capable of metastasis formation and producing low ROS level as a homogeneous population. Tumor spheres characterized by suspension growth in culture medium could efficiently maintain CSCs without restriction to a particular surface phenotype and have been widely used to enrich CSCs in cancer research [11, 23–25]. In this study, we isolated tumor spheres from 4 different cancer cell lines including 4 T1, HT29, HCT116, and SW480, and investigated the correlation between ROS level of tumor spheres and cancer metastasis. The objectives of our study are: (1) To determine whether the tumor spheres are composed of a homogeneous population with lower ROS level as compared to non-cancer stem cells? (2) To examine what are the underlying mechanisms of ROS in regulating cancer metastasis?

## Methods

### Cell culture

Four cell lines (4 T1, SW480, HCT116 and HT29) were purchased from the Cell Bank of Type Culture Collection of the Chinese Academy of Sciences (Shanghai, China). 4 T1 cells were maintained in complete RPMI-1640 (Life Technologies). SW480 cells were cultured in Leibovitz’s L-15 medium. HCT116 and HT29 cells were maintained in complete McCoy’s 5a Medium. Culture mediums were all supplemented with 10% FBS, 100 U/mL penicillin, and 100 μg/mL streptomycin, and 2 mM L-glutamine in a 5% CO_2_ environment at 37 °C. The authentication testing of cell lines have been performed by Shanghai Biowing Applied Biotechnology Co., Ltd. All the cell lines used in our study matched the standards recommended for cell lines authentication. All the cells were mycoplasma-free and routinely checked with the PCR Mycoplasma detection kit (K0103, HuaAn Biotechnology).

### Establishment of tumor spheres, ROS-high tumor spheres (RH-TS) and ROS-low tumor spheres (RL-TS)

To induce tumor spheres in 4 T1, SW480, HCT116, and HT29 cell lines, cells were dissociated enzymatically using 0.05% Trypsin-EDTA solution, diluted to a density of 2 × 10^4^/mL, and then plated in non-coated petri dishes. Cells were grown in serum-free DMEM/F12 medium containing 40 ng/mL basic fibroblast growth factor (bFGF) and 20 ng/mL epidermal growth factor (EGF) (both from PeproTech), B-27® Serum-Free Supplement (17,504,044, Gibco), N-2 Supplement (17,502,048, Gibco), and 4 μg/mL Heparin sodium salt (H3393, Sigma-Aldrich). The cells were cultured in an incubator at 37 °C with 5% CO_2_. Tumor spheres collected after ~ 14 days culture were used for further study. We further measured the cellular ROS level represented by DCFH-DA of tumor spheres of 4 T1 cells, HCT 116 cells, SW480 cells and HT29 cells using confocal microscope and flow cytometry. We found that tumor sphere cells were composed by two major subpopulations of cells on the basis of ROS concentration, with the ROS high-tumor spheres cells (RH-TS) and ROS low-tumor spheres cells (RL-TS). We used flow cytometry to separate and collect RH-TS cells and RL-TS cells for further studies.

### 3-D culture, frozen section, immunofluorescence, or hematoxylene and eosin staining

We used the 3 Dimensions (3D) on-top assay to analyze the colony morphology of RH-TS cells and RL-TS cells. Generally, the 8-well glass with pre-chilled culture surface (Millicell EZ SLIDE 8-well glass, PEZGS 0816) was covered with 150 μL per well Matrigel™ Basement Membrane Matirx (BD 356234), incubated for 15 min at 37 °C to allow the Matrigel to solidify. Cells at a concentration of ~ 100 cells/well were cultured in the 8-well glass with the Matrigel-medium mixture (10% volume of Matrigel) to the plated culture and the mixture was replaced every 2 days. After 10–14 days culture, the 3D cell clones were harvested for further analysis. For frozen section, the 3D clones were embedded in O.C.T. Compound (4583, SAKURA Tissue-Tek). Five μm sections were cut on a cryostat (Leica CM1950; Leica, Wetzlar, Germany) and post-fixed in ethanol: acetone fixation solution (with a volume of 3:1 ethanol: acetone) for 10 min at room temperature. For immunofluorescence, frozen sections were permeabilized with 0.2% Triton X-100 for 15 min, blocked with 3% BSA for 1 h and incubated with anti-EpCAM antibody (1:1000 dilution of ab71916, Abcam, UK) or anti-TP63 antibody (1:50 dilution of ab735, Abcam, UK) according to the protocols of the suppliers. All post-fixed sections were mounted and viewed under Zeiss LSM 710 laser confocal microscope (Carl Zeiss, Jena, Germany). Moreover, frozen sections stained with hematoxylene and eosin (HE) were evaluated and photographed with a Nikon Eclipse 50i microscope equipped with NIS-Elements software (version 3.22).

### Drug-resistance analysis

Tumor sphere cells (red fluorescence, loaded with 10 μM SNARF™-1) and parental cells (green fluorescence, loaded with 5 μM CFDA SE) were treated with drugs including cisplatin (5 μg/L), methotrexate (MTX, 10 μg/L), doxorubicin (Dox, 10 μg/L), taxel (50 μg/L), Etoposide (10 μg/L), or VCR (5 μg/L). After 2 days of incubation, the cells were observed under a Zeiss LSM710 laser confocal microscope (Carl Zeiss, Germany) equipped with Zen software to process the images.

### Immunofluorescence

For immunofluorescence of tumor cells, 4 T1 cells grown on cover slip and primary tumor spheres which were isolated from 4 T1 parental cells and then centrifuged onto glass slides were fixed with 3.7% paraformaldehyde for 15 min and permeabilized with 0.2% Triton X-100 for 15 min at room temperature. The cells were blocked with 3% BSA for 1 h and incubated with anti-CD24 antibody (1:100 dilution of ab25657), anti-CD44 antibody (1:100 dilution of ab119863), anti-EpCAM antibody (1:1000 dilution of ab71916), anti-TP63 antibody (1:50 dilution of ab735), anti-ASMA antibody (1:200 dilution of ab3280), or anti-CD49f antibody (1:100 dilution of ab95703), according to the protocols of the suppliers. Nuclei were counterstained with DAPI. All antibodies were purchased from Abcam. All post-fixed sections were mounted and viewed under Zeiss LSM 710 laser confocal microscope (Carl Zeiss, Jena, Germany) equipped with Zen software to process the image.

For immunofluorescence of tumor tissues, anti-EpCAM and anti-TP63 antibodies were used to evaluate the differentiation of RH-TS cells in the tumor samples. Briefly, tumor tissue sections were permeabilized with 0.2% Triton X-100 for 15 min, blocked with 3% BSA for 1 h and incubated with anti-EpCAM antibody (1:1000 dilution of ab71916, Abcam, UK) or anti-TP63 antibody (1:50 dilution of ab735, Abcam, UK) according to the protocols of the suppliers. All post-fixed sections were mounted and viewed under Zeiss LSM 710 laser confocal microscope (Carl Zeiss, Jena, Germany).

### Measurement of cellular reactive oxygen species

Cellular ROS was detected using specific ROS probes by confocal microscope and flow cytometry. For flow cytometric measurement, cells were incubated with 2′,7′-Dichlorodihydrofluorescein diacetate (DCFH-DA, 10 μM, Sigma, D6883) or dihydroethidium (DHE, 5 μM, Sigma, D7008) for 30 min at 37 °C, after which the cells were washed and suspended in ice-cold PBS and analyzed for fluorescence intensity using a 485 nm excitation beam (FACS ArrayBioanalyzer, BD biosciences). Flow Jo 7.6 was utilized to quantify the mean fluorescence intensity. For confocal measurement, ROS measurement was assayed by DCFH-DA, DHE, MitoSOX TM Red mitochondrial superoxide indicator (MitoSOX Red, Invitrogen, M36008), or 4-amino-5-methylamino-29,79-difluorofluorescein (DAF-FM, Sigma, D2321), according to manufacturer’s instruction. Briefly, cells were loaded with DCFH-DA (10 μM for 30 min), DHE (5 μM for 30 min), MitoSOX Red (2.5 μM for 10 min), or DAF-FM (10 μM for 30 min), washed with ice cold HBSS (Hank’s Balanced Salt Solution, pH 7.2), and then observed under a Zeiss LSM710 laser confocal microscope (Carl Zeiss, Germany) equipped with Zen software to process the images.

### In vivo metastatic assay

BALB/c female mice and nude mice (4–5 weeks old) were obtained from animal experimental center of Zhejiang University of Traditional Chinese Medicine. Nude mice were housed in a standard polypropylene cage, housed in groups of five and given five days to acclimate to the housing facility. Environmental conditions were maintained at a temperature of 21 °C ± 2 °C, humidity of 55% ± 10%, and a 12:12 light:dark cycle. Animals were housed in 595 × 380 × 200 mm cages (Techniplast UK, 1354G Eurostandard Type IV) with food and water provided ad libitum. During housing, animals were monitored twice daily for health status. No adverse events were observed.

BALB/c mice were used to analyze 4 T1 cells, and nude mice were used to analyze the other three types of cancer cells. Mice were randomly inoculated with parental cells (~ 10,000 cells per mouse), tumor sphere cells (~ 10,000 cells per mouse), RH-TS cells (~ 10,000 cells per mouse), or RL-TS cells (~ 10,000 cells per mouse). Tumor spheres, RH-TS, and RL-TS were dissociated enzymatically using 0.05% Trypsin-EDTA solution, and counted. To analyze the differentiation ability of 4 T1 tumor sphere in vivo and avoid the influence of the murine breast tissue on cell differentiation, we inoculated one tumor sphere per Balb/C mouse subcutaneously. To analyze the cancer metastasis ability of tumor sphere in vivo, 4 T1 cells and its derived tumor spheres, RH-TS and RL-TS cells were orthotopically inoculated into the mammary fat pad of female BALB/c mice, and HCT116 cells, SW480 cells, HT29 cells and their derived tumor sphere, RH-TS and RL-TS cells were inoculated subcutaneously into the right flank of nude mice. Tumor volume was calculated using the formula V = (L × W^2^) × 0.5, where L and W equal mid-axis length and width, respectively. On the day of sacrifice, the mice were anesthetized with penobarbital sodium, pulmonary metastases were scored by counting the macroscopic metastatic nodules. For hepatic metastasis, sections were stained with H&E and examined microscopically. The incidence of hepatic metastasis was quantified by counting the total tissue area per liver section (D1) and metastasis present in the same area (D2). The metastatic index was calculated by the ratio D2/D1 [26]. The relative area of metastases was measured using the Image J 1.45S software (NIH). All procedures were carried out in accordance with the Animals (Scientific Procedures) Act, 1986 (UK) (amended 2013). A completed ARRIVE guidelines checklist is included in Additional file 1: Checklist S1.

### Transcriptome sequencing

The whole expression files of 4 T1 parental cells, RH-TS cells, and RL-TS cells were investigated by using mRNA-Seq experiments performed by Novogene (Beijing, China). mRNA-seq library is prepared for sequencing using standard Illumina protocols. Gene Ontology (GO) enrichment analysis of differentially expressed genes was implemented by the cluster Profiler R package, in which gene length bias was corrected. GO terms with corrected *P* value less than 0.05 were considered significantly enriched by differential expressed genes. The statistical enrichment of differential expression genes in KEGG pathways was performed by the cluster Profiler R package.

### Real-time PCR

To validate the gene chip data, we determined the expression of the following genes with real-time PCR. The procedure is described in detail in the Supporting information, Supplementary materials and methods.

### Western blot analysis

The procedure is described in detail in the Supporting information, Supplementary materials and methods.

### Calculation of NAD/NADH ratio

We measured the free NADH/NAD+ ratio as previously described by Sun F et al. [27]. Briefly, the cytosolic lactate and pyruvate concentration were determined by HPLC. NAD/NADH was estimated by the L/P ratio and the equation of the chemical equilibrium which is reported by Williamson et al. Apparent Keq = [pyruvate] eq [NADH] eq. [H+]/ ([Lactate] eq [NAD]eq) = 1.11 X 10^− 11^, where pH is 7.0, If LDH catalyzed reaction is allowed to proceed to equilibrium, the final products and reactants could be expressed by the equation: Apparent Keq = Keq [H+], where Keq = [pyruvate] eq [NADH]eq/([Lactate] eq [NAD]eq) = 1.11 X 10^− 4^, or [NAD]/[NADH] = [pyruvate]eq/(Keq [Lactate]eq).

### Transmission electron microscopy

Cells were fixed in 2.5% glutaraldehyde in 0.1 M sodium phosphate buffer (pH 7.4) overnight. The samples were treated with 1.5% osmium tetroxide, dehydrated with acetone, embedded in durcupan resin, post-stained with lead citrate and examined in a TECNAI 10 electron microscope (Philips, The Netherlands) at 60 kV.

### Survival analysis

Kaplan-Meier plots summarized results from analysis of correlation between mRNA expression level and patient survival from Human Protein Atlas database (http://www.proteinatlas.org), using best separation. Patients were divided based on level of expression into one of the two groups “low” or “high”. The 5-year survival for patients with high expression, 5-year survival for patients with low expression and log-rank *P* value are displayed. For glioma, 3-year survival is shown.

### Statistical analysis

All data were analyzed using the InStat software (GraphPad, CA, USA) and displayed as mean ± SD. Two-tailed Student’s t-test was used for statistical analysis, and significance was defined at *** *p*<0.001, ** *p*<0.01, * *p*<0.05.

## Results

### The cancer stem cell features of tumor spheres

**Fig. 1:**
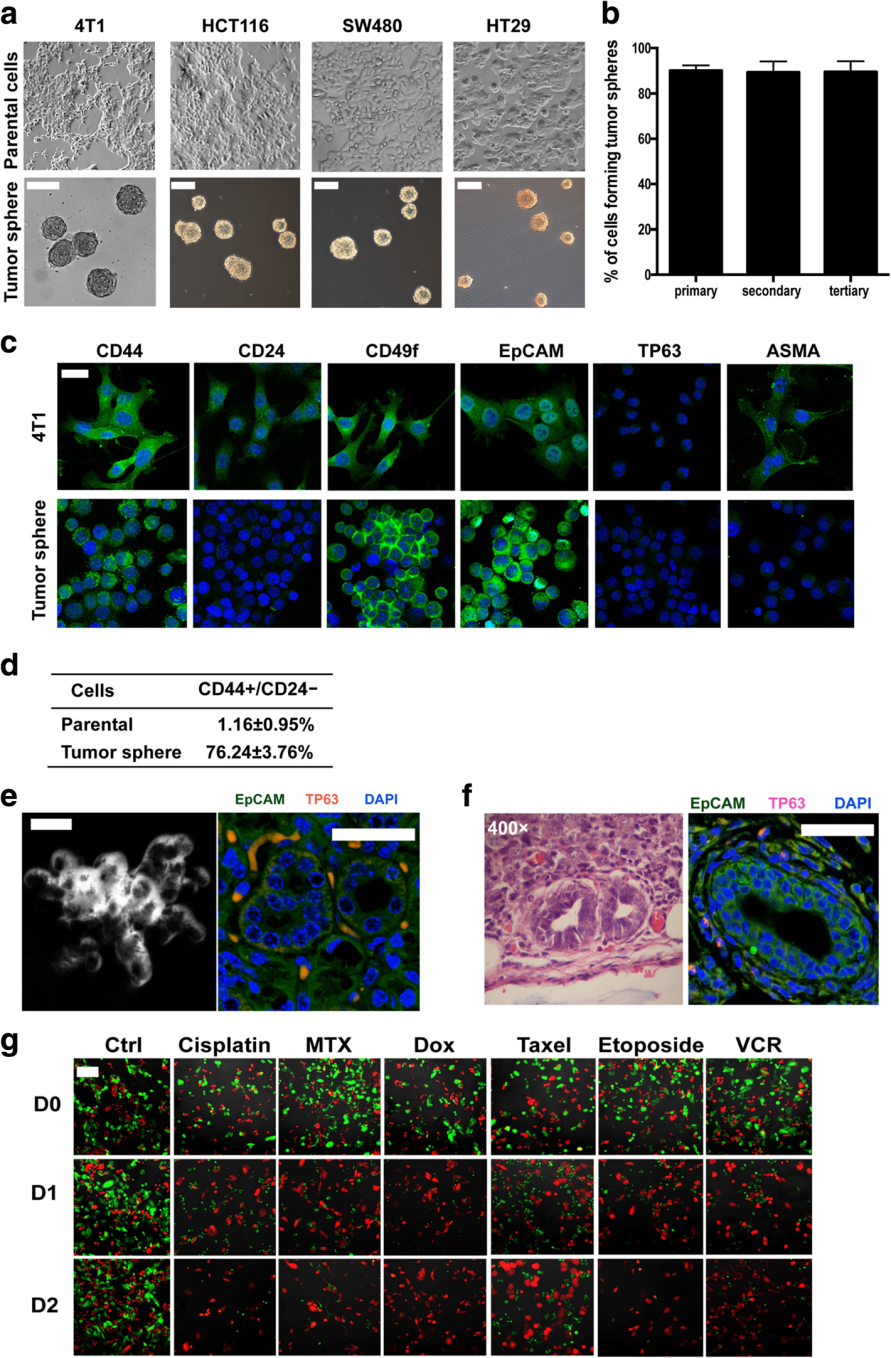
Tumor sphere cells possessed CSCs features. **a** Tumor spheres were isolated from 4 T1 cells, HCT116 cells, SW480cells, and HT29 cells by using cancer stem cell culture method as described in Experimental Procedures. Scale bars represent 100 μm. **b** The relative proportion of tumor sphere cells remained stable after 3 generations of replating. Results are shown as mean values ± SEM from three independent experiments. **c** The surface markers of 4 T1 parental cells and primary tumor sphere cells isolated from 4 T1 parental cells. Scale bars represent 50 μm. **d** The percentage of CD44 + CD24- cells in 4 T1 parental cells and tumor sphere cells. Results are from three independent experiments. **e** 4 T1 tumor sphere cells grown in 3D in matrigel formed acinar-like colony (a representative image under bright field, phase contrast microscopy) and differentiation markers (TP63, myoepithelial cell marker; EpCAM, epithelial cell marker). Data shown are representative of three independent experiments. **f** Tumors from tumor sphere cells containing glandular tubules stained with hematoxylin/eosin (H&E) and differentiation markers (TP63, myoepithelial cell marker; EpCAM, epithelial cell marker). Data shown are representative of three independent experiments. **g** Co-culture of tumor sphere cells (red fluorescence) and 4 T1 cells (green fluorescence) with drugs including cisplatin, methotrexate (MTX), doxorubicin (Dox), taxel, etoposide, or vincristine (VCR). After 2 days of incubation, much more tumor sphere cells survived than 4 T1 cells. Results are representative of three independent experiments. Scale bars =100 μm

We isolated tumor spheres from 4 T1, HCT116, SW480, and HT29 cells in vitro (Fig. 1a) and assessed the proliferation, self-renewal potential, differentiation and drug-resistance of tumor sphere cells quantitatively. In 4 T1 cells, 50 tumor spheres were trypsinized and counted by using trypan blue exclusion assay. Each single tumor sphere contained 1085 ± 325 cells. We further replated dissociated single tumor sphere cells in a 96-well plate in an average density of 1 cell per well to analyze the self-renewal ability. 3833 single clones were obtained from tumor sphere 1° cells from 20 clones, and the percentages of tumor sphere was 90.1% ± 1.3%; 747 single clones were obtained from tumor sphere 2° cells from 5 clones, and the percentages of tumor sphere was 89.3% ± 1.1%; 673 single clones were obtained from tumor sphere 3° cells from 5 clones, and the percentages of tumor sphere was 89.5% ± 1.6% (tumor sphere 1°, tumor spheres generated from 4 T1 cells; tumor sphere 2°, tumor spheres generated from tumor sphere 1° cells; tumor sphere 3°, tumor spheres generated from tumor sphere 2° cells). Thus, it revealed that about 90% of sphere derived single cells grew into new tumor spheres, and the percentage of self-renewing sphere-forming cells remained remarkably stable over 3 rounds of single cell replating experiments (Fig. 1b). Tumor sphere cells giving rise to large numbers of daughter cells were able to form spheres in vitro and thereby defined as self-renewal.

We then analyzed the surface markers of tumor sphere cells by immunofluorescence with a panel of markers according to Pece et al. [28]. The tumor sphere cells isolated from 4 T1 parental cells were CD44 + CD24-, while parental cells were CD44 + CD24+ (Fig. 1c). Moreover, tumor sphere cells expressed higher CD49f and lower ASMA than parental cells, while both parental cells and tumor sphere cells were EpCAM+TP63- (Fig. 1c). We further measured the expression of CD24 and CD44 by sorting. Most tumor sphere cells were CD44 + CD24-, while only 1.16 ± 0.95% parental cells were CD44 + CD24- (Fig. 1d). It is worthy pointing out that both parental cells and tumor sphere cells were not homogenous because the labeling intensity differed markedly, such as the CD44 and CD24 fluorescent intensity. Increasing evidence has demonstrated that Lgr5 marks a population of colon cancer CSCs. To study whether tumor spheres isolated from colon cancer cells possessed CSCs features, we examined the Lgr 5 expression in tumor spheres. As shown in Additional file 2: Figure S1, tumor spheres of HCT116 cells, SW480 cells, and HT29 cells were all Lgr 5^+^.

We next used 3D culture methods to analyze the differentiation ability of tumor spheres in vitro. Under 3D culture, tumor sphere cells formed acinar-like colonies containing both epithelial (EPCAM positive cells) and myoepithelial cells (TP63 positive cells) (Fig. 1e). To analyze the differentiation ability of 4 T1 tumor sphere in vivo and avoid the influence of the murine breast tissue on cell differentiation, we inoculated one tumor sphere per Balb/C mouse subcutaneously. It showed that xenotransplantation of tumor spheres subcutaneously reproducibly formed tumors. Of note, the mammary epithelium-like structure with differentiation markers (both EPCAM and TP63) was observed in the xenografts generated by tumor sphere cells (Fig. 1f). These data suggested that tumor sphere cells have the differentiation ability in vitro and in vivo.

Furthermore, we measured the drug-resistant ability of tumor spheres. As shown in Fig. 1g and Additional file 2: Figure S2, tumor sphere cells labeled with SNARF™-1 (red fluorescence) were obviously more resistant to anticancer drugs, such as cisplatin, methotrexate (MTX), doxorubicin (Dox), etoposide and vincristine (VCR) than parental cells labeled with CDFA SE (green fluorescence).

We also sorted out ROS^high^ cells with the same ROS level represented by DCFH-DA as RH-TS cells from 4 T1 parental cells by FCM (Additional file 2: Figure S3a). Using limiting dilution assay, we observed that ROS^high^ cells produced significantly more tumor sphere clones than ROS^low^ cells, but significantly less tumor sphere clones than RH-TS cells (Additional file 2: Figure S3b). The results suggested that ROS^high^ cells isolated from parental cells also had some feature of CSCs.

Taken together, our data revealed that tumor spheres possessed the characteristic features of CSCs, such as a high capacity of self-renewal, differentiation, and drug-resistance.

### RH-TS exclusively drive metastases formation

**Fig. 2:**
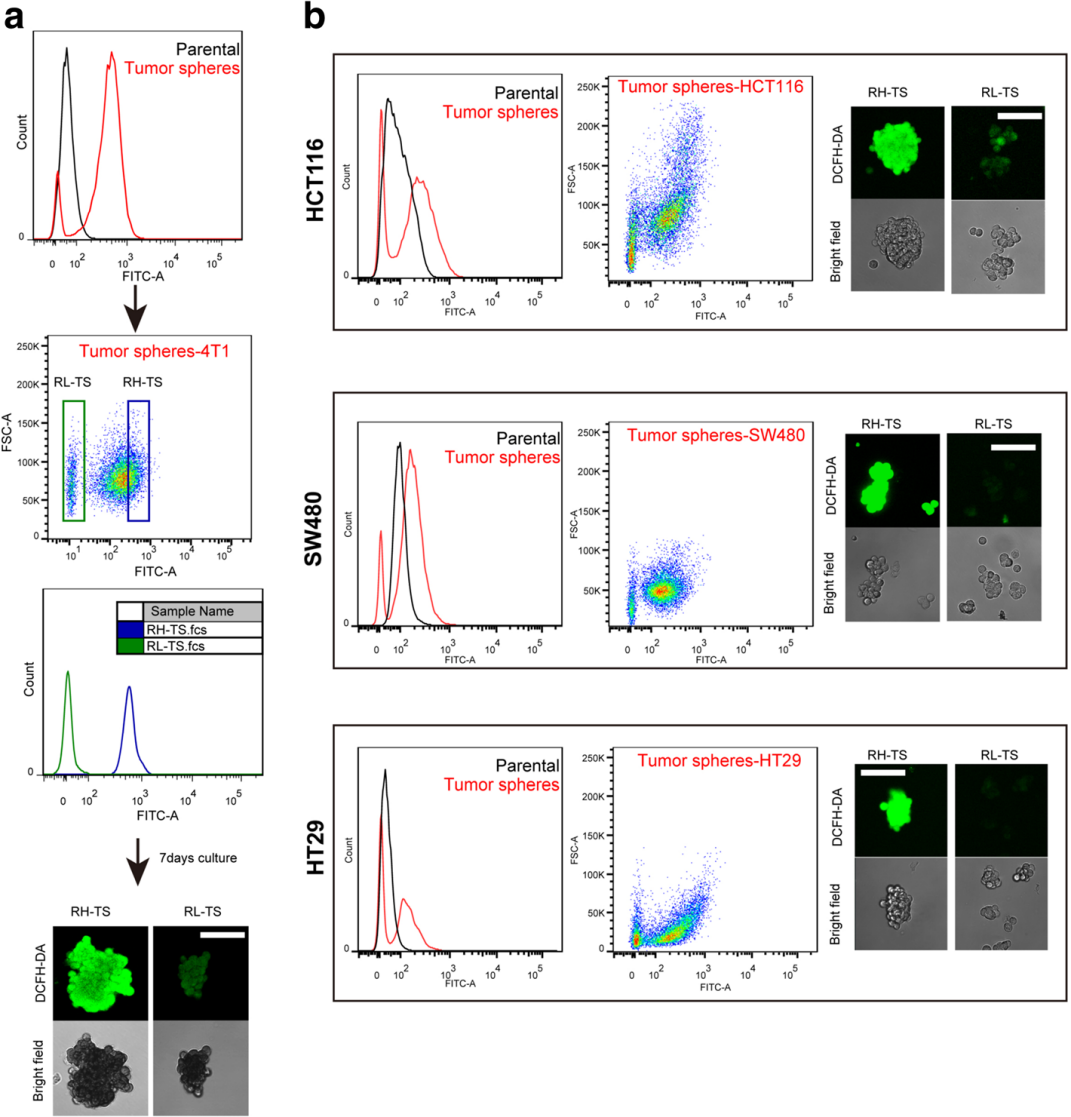
Isolation of RH-TS and RL-TS cells. **a** RH-TS and RL-TS cells were isolated from 4 T1 tumor spheres by flow cytometry and intracellular ROS concentrations were measured by DCFH-DA staining. RH-TS and RL-TS cells were re-analyzed by FACS and confocal microscopy. **b** Tumor spheres were isolated from HCT116 cells, SW480 cells, and HT29 cells. ROS profile representing by DCFH-DA fluorescence was measured by FACS and confocal microscopy. Scale bars =100 μm

We further measured the cellular ROS level of tumor spheres, which was probed by DCFH-DA and detected via confocal microscope and flow cytometry. As shown in Fig. 2, tumor sphere cells were composed by two major subpopulations of cells on the basis of ROS concentration, with the ROS high-tumor spheres (RH-TS) being significantly over-represented as compared to ROS low-tumor spheres (RL-TS).

**Fig. 3:**
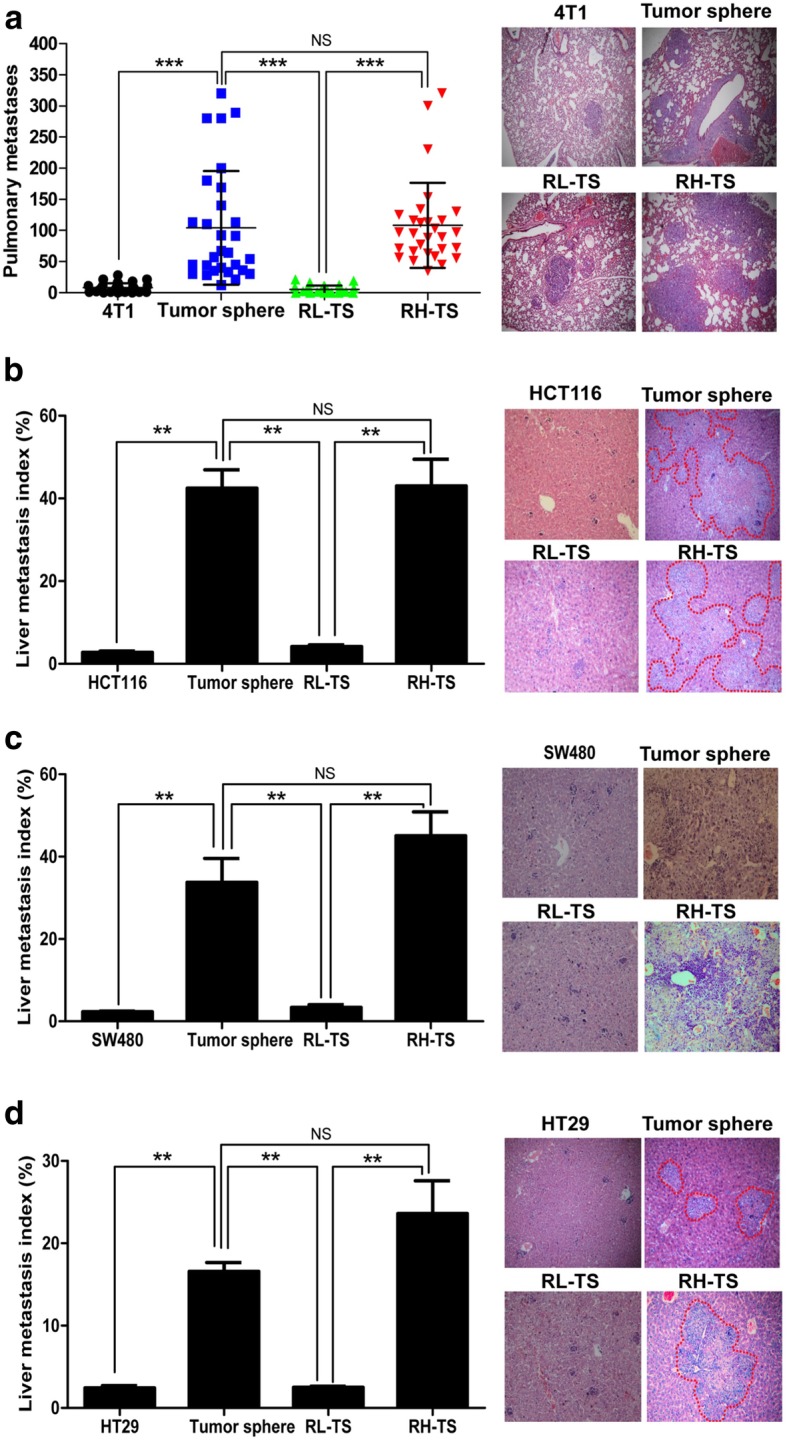
RH-TS contributed to In Vivo cancer metastasis. **a** The pulmonary metastases of tumor spheres and RH-TS were much more than that of 4 T1 cells and RL-TS when orthotopically inoculated into Balb/c mouse. Five independent experiments. NS, not significant; ***, *p* < 0.001. **b** The liver metastases of tumor spheres and RH-TS were much more than that of HCT116 cells and RL-TS when inoculated subcutaneously into NOD/SCID mouse. Two independent experiments performed. NS, not significant; **, *p* < 0.01. **c** The liver metastases of tumor spheres and RH-TS were much more than that of SW480 cells and RL-TS when inoculated subcutaneously into NOD/SCID mouse. Two independent experiments performed. NS, not significant; **, p < 0.01. **d** The liver metastases of tumor spheres and RH-TS were much more than that of HT29 cells and RL-TS when inoculated subcutaneously into NOD/SCID mouse. Two independent experiments performed. NS, not significant; **, p < 0.01

To analyze the metastatic ability of tumor spheres and identify the metastatic cancer cells, we inoculated 4 T1 cells, tumor spheres cells, RH-TS cells or RL-TS cells orthotopically into mammary fat pads of Balb/c mice. Tumor spheres induced much more pulmonary metastases than 4 T1 cells. Significantly more pulmonary metastases were observed in the lung tissue of mice inoculated with RH-TS cells than those of mice inoculated with RL-TS cells. No difference was found in pulmonary metastases between mice received RL-TS cells and mice received 4 T1 cells (Fig. 3a, Additional file 2: Figure S4). We further evaluated the metastatic ability of RH-TS in HCT116, SW480 and HT29 cells in NOD-SCID mice subcutaneously. We obtained consistent results (Fig. 3b-d, Additional file 2: Figure S5-S7) that RH-TS cells highly metastasized to the liver while RL-TS cells exhibited low metastatic ability.

We have demonstrated that hydrogen peroxide (H_2_O_2_) induced a high oxidative stress but permits cancer cells growth [29]. Recently, Wang et.al reported that cellular ROS promoted cancer cell metastasis in vitro [30]. To clarify whether ROS promote cancer cell invasion, we used Transwell invasion assays to analyze the invasion ability of 4 T1 cells with or without H_2_O_2_. As shown in Additional file 2: Figure S8, 4 T1 cells exhibited higher invasion ability with incubation of 0.1 mM H_2_O_2_.

Collectively, our data showed that tumor spheres have higher capacity of metastasis compared to the parental cells, in which RH-TS cells are the exclusive cell population responsible for the metastasis.

### Level of mitochondrial ROS is elevated significantly in RH-TS

**Fig. 4:**
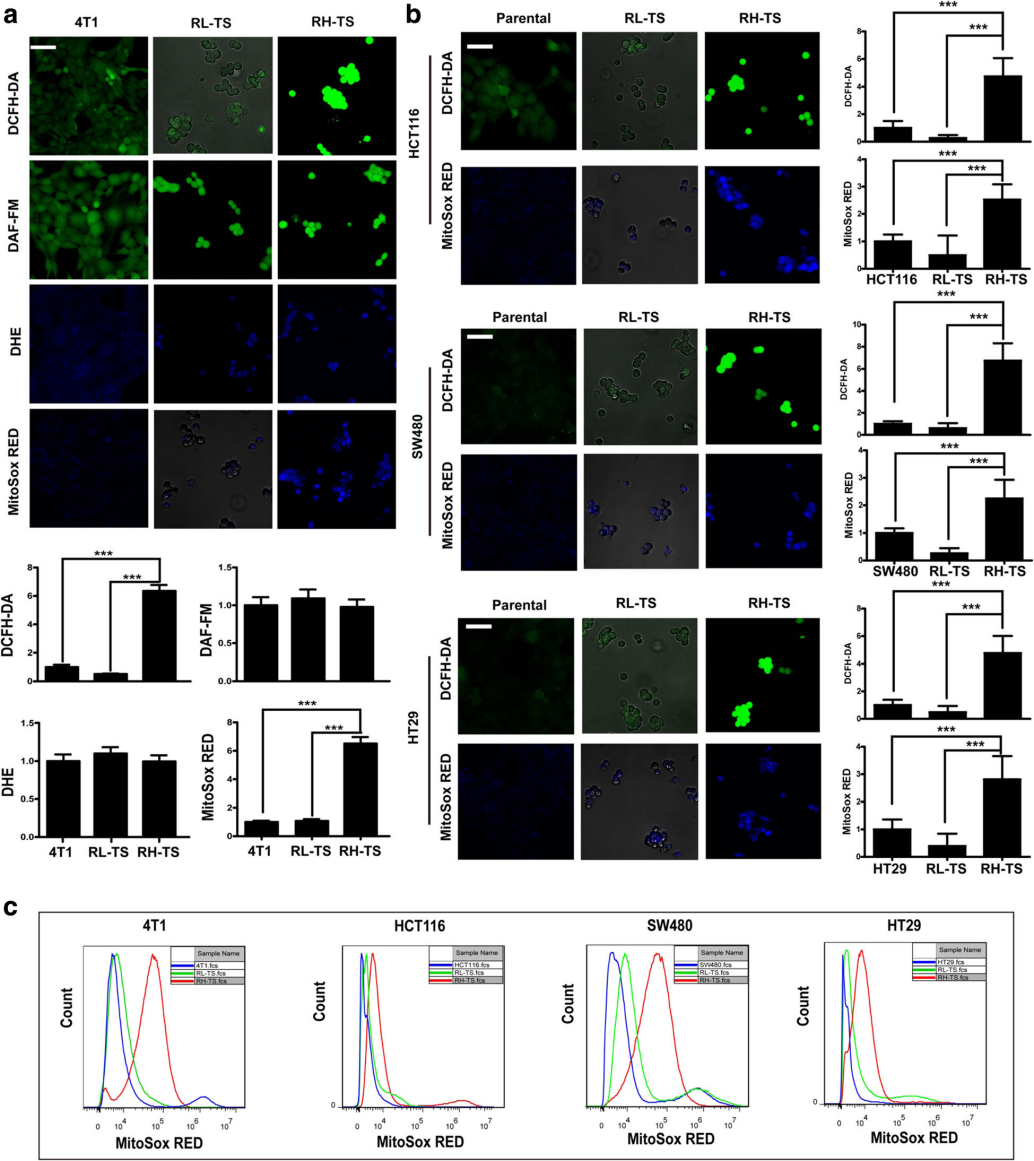
Mitochondrial ROS elevated significantly in RH-TS. **a** After isolation of RH-TS and RL-TS cells from 4 T1 tumor spheres, the ROS composition was measured by DCFH-DA, DAF-FM, DHE, and MitoSox Red as described in Materials and Methods. Scale bar = 50 μm. *** p < 0.001. **b** The ROS composition of RH-TS and RL-TS cells in HCT116, SW480, and HT29 cells was measured by DCFH-DA, DHE and MitoSOX Red. Results are shown as mean ± SEM from three independent experiments. Scale bar = 50 μm. *** p < 0.001. **c** RH-TS cells increased MitoSox Red level significantly. MitoSox Red levels of parental cells (blue), RL-TS (green), RH-TS (red) were analyzed by flow cytometry. Three independent experiments performed

We first analyzed the ROS composition of RH-TS and RL-TS using ROS specific probes by confocal microscope as described previously [29]. We found that total ROS concentrations (DCFH-DA fluorescence) and intra-mitochondrial superoxide (MitoSOX Red fluorescence) in RH-TS were higher than that in RL-TS, whereas the extra-mitochondria superoxide level (DHE fluorescence) and Nitric oxide level (DAF-FM fluorescence) remained equal in 4 T1 cells (Fig. 4a). Similar results were obtained among HCT116 cells, SW480 cells and CT29 cells (Fig. 4b). Moreover, we measured the intra-mitochondrial superoxide levels by flow cytometry. As shown in Fig. 4c, markedly increased intra-mitochondrial superoxide level was observed in RH-TS cells compared to that in parental cells and RL-TS cells. These results suggested that the majority of endogenous ROS of RH-TS was generated in mitochondria.

### Metabolic remodeling of RH-TS contributes to an elevated mitochondrial ROS level

**Fig. 5:**
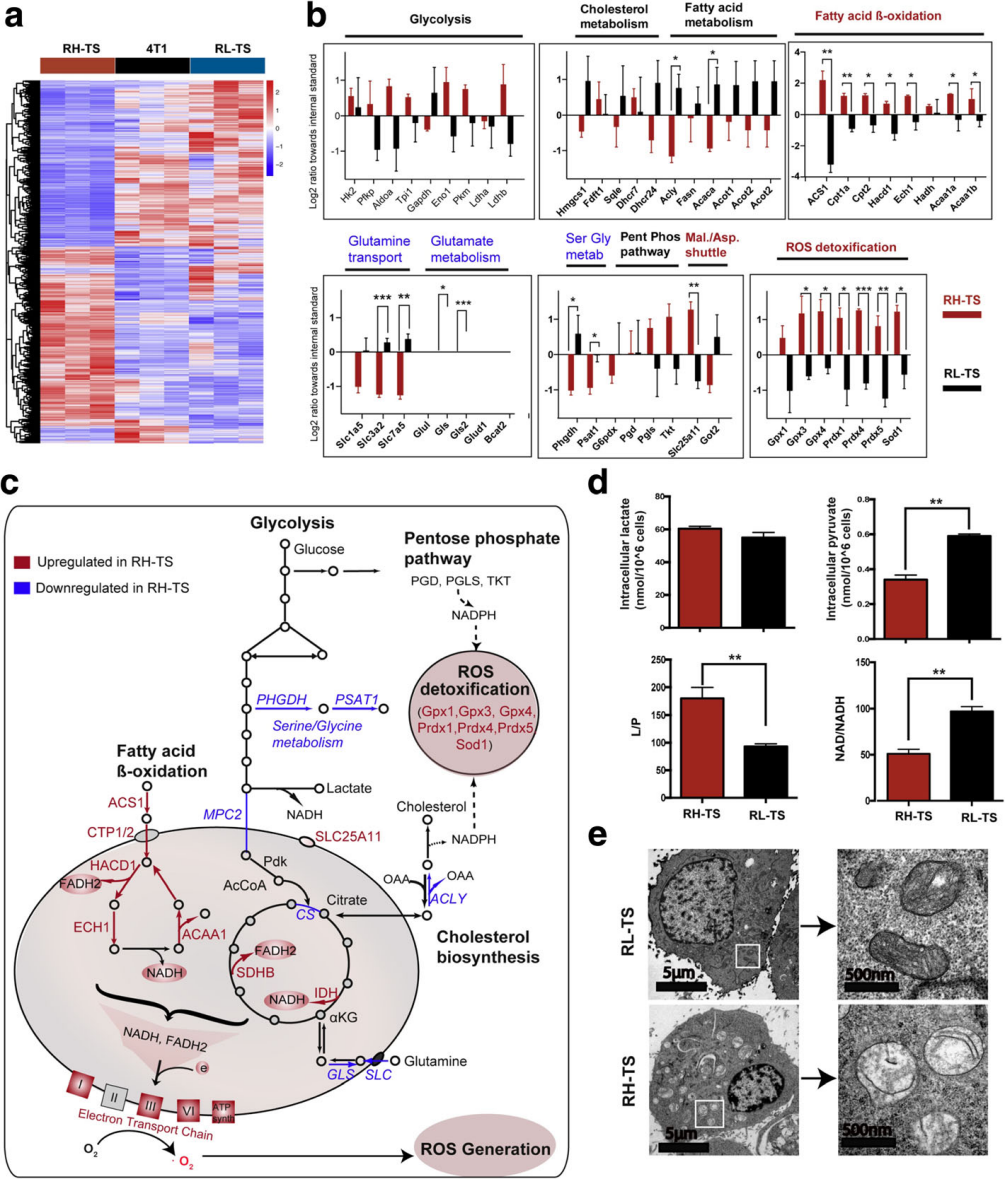
Metabolic remodeling in RH-TS. **a** Heatmap of differentially expressed genes among parental cells, RH-TS cells, and RL-TS cells. **b** Expression changes of metabolic enzymes between RH-TS cells and RL-TS cells. * *p* < 0.05, ** p < 0.01, *** p < 0.001; unmarked, not significant. **c** A diagram depicting the changes in major metabolic pathways in RH-TS cells compared to RL-TS cells. Red objects and blue objects represent significant upregulation or downregulation in RH-TS cells, respectively. **d** The cytosolic free NAD/NADH ratio in RH-TS cells was significantly less than that in RL-TS cells. **e** Representative photographs of cell mitochondria by transmission electron microscopy

Mitochondrial electron transport chain (ETC) is the important source of ROS within most mammalian cells. The generation of ROS in mitochondria is largely dependent on the NADH/NAD^+^ [31], which is correlated with cell metabolism. To investigate the expression of genes involved cell metabolism among parental cells, RH-TS cells, and RL-TS cells, we performed RNA-seq analysis and found that RH-TS cells upregulated 53 genes and downregulated 59 genes compared with both parental and RL-TS cells (Fig. 5a, Additional file 2: Table S1-S2). We zoomed in on the entire metabolic network to elucidate the individual regulated pathways (Fig. 5b-c). Our results suggested reduced biosynthesis and increased breakdown of fatty acids in RH-TS cells. The key enzyme, ATP-citrate lyase (ACLY) that produces acetyl-CoA for lipid and cholesterol biosynthesis was significantly downregulated in RH-TS. However, enzymes such as ACS1, CPT1/2, HADC1, ECH1, and ACAA1 in fatty acid β-oxidation pathway were significantly elevated, pointing to potential utilization of this pathway to generate reducing power in the form of NADH (Fig. 5c). Moreover, the enzymes in TCA cycle (such as IDH and SDHB, which alongside with high production of NADH and FADH2), were highly upregulated. Furthermore, the component of ETC, such as ND1/2/3/4/6, Ndufa4/5, Ndufb4, COX3/4/5B/6A/7/8, and Cyt1/b, were also significantly upregulated. Moreover, there was no significant change in the metabolic pathways including glycolysis and the pentose phosphate pathway (PPP) whereas the serine/glycine biosynthetic pathway was downregulated primarily due to the significant reduction in expression of PHGDH and PSAT1. The cytosolic free NAD/NADH ratio in RH-TS cells was significantly less than that in RL-TS cells (Fig. 5d). We further identified the mitochondrial morphologies based on observation of electron micrographs. As shown in Fig. 5e, the mitochondrion in RH-TS cells showed swollen structures while RL-TS cells showed dense structure. Concurrently, to maintain the redox homeostasis of RH-TS cells, ROS detoxification pathways were significantly upregulated with increased glutathione peroxidase (GPX)-3 and GPX-4, peroxiredoxin (PRDX)-1, PRDX-4, and PRDX-5, and superoxide dismutase (SOD)-1. Taken together, gene expression profiles suggested that increased breakdown and reduced synthesis of fatty acids might be the source of increased mitochondrial ROS.

### MAPK cascades and the epithelial-mesenchymal transition are predicted to be downstream of elevated mitochondrial ROS

**Fig. 6:**
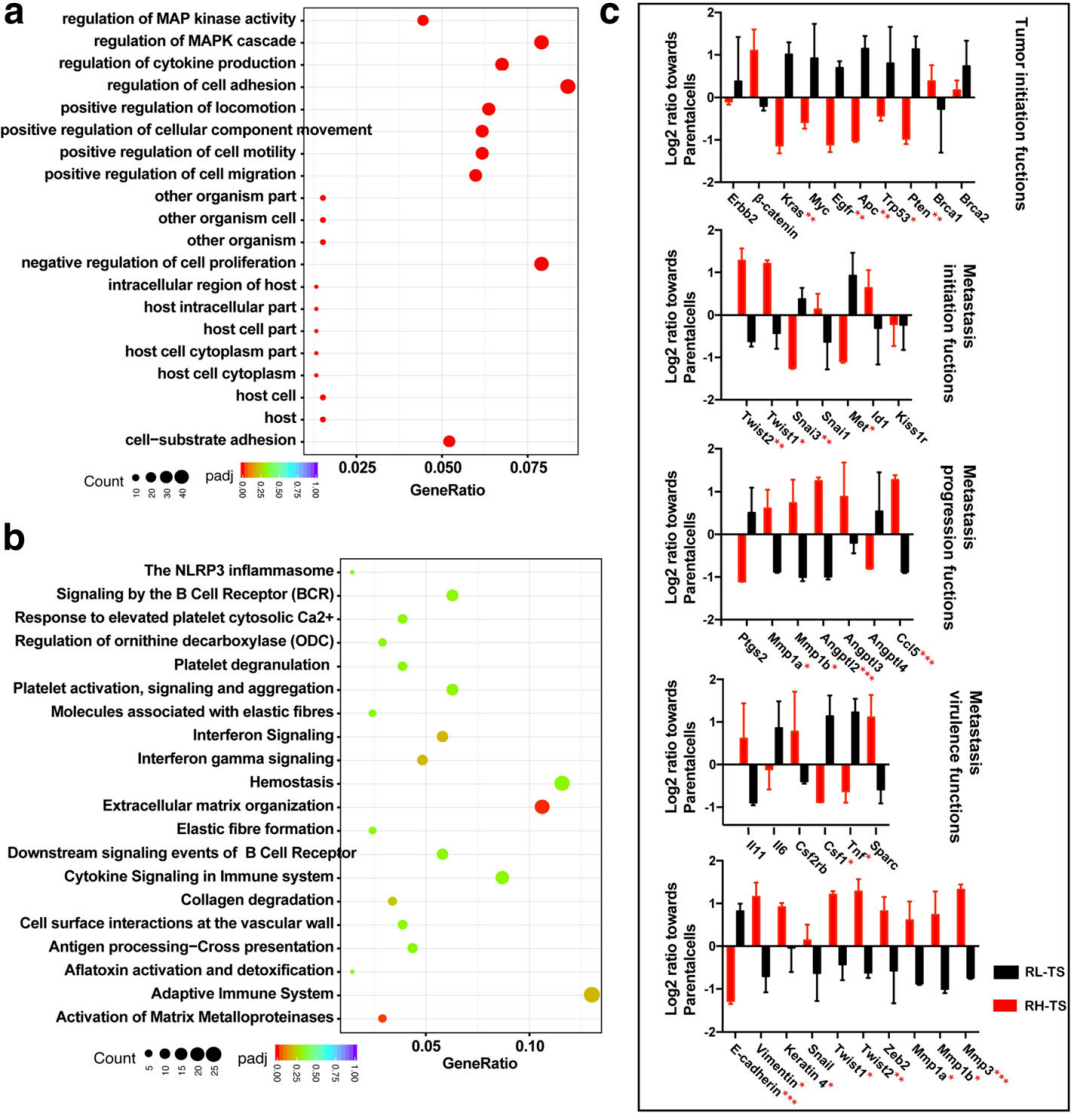
MAPK signaling and genes for cell invasion and metastasis in RH-TS. **a** The GO enrichment analysis of differentially expressed genes between RH-TS cells and RL-TS cells. **b** The reactome analysis of differentially biological pathways between the RH-TS cells and RL-TS cells. **c** The metastasis genes expression of RH-TS and RL-TS. * p < 0.05, ** p < 0.01, *** p < 0.001

Excessive ROS can potentiate tumor progression and stimulate cell invasion through redox regulation of ROS-dependent pathways including the mitogen-activated protein kinase (MAPK) cascades, the phosphoinositide 3-kinase (PI3K) pathway, and nuclear factor kappa light chain enhancer of activated B cells (NF-κB) pathway [22, 32–34]. As shown in Fig. 6a-b, the GO enrichment and reactome analysis showed a large cluster in MAPK signaling pathway (regulation of MAP kinase activity, regulation of MAPK cascade) and cell invasion and metastasis (regulation of cell adhesion, positive regulation of locomotion, positive regulation of cellular component movement, positive regulation of cell motility, positive regulation of cell migration, cell-substrate adhesion, extracellular matrix organization and activation of matrix metalloproteinases). Moreover, we detected the metastasis genes as described by Nguyen et al. [35]. RH-TS expressed significantly higher levels of TWIST1, TWIST2, SNAI1, SNAI2, MMP1, MMP3 and LOX, but significantly lower levels of DARC (Fig. 6c).

**Fig. 7:**
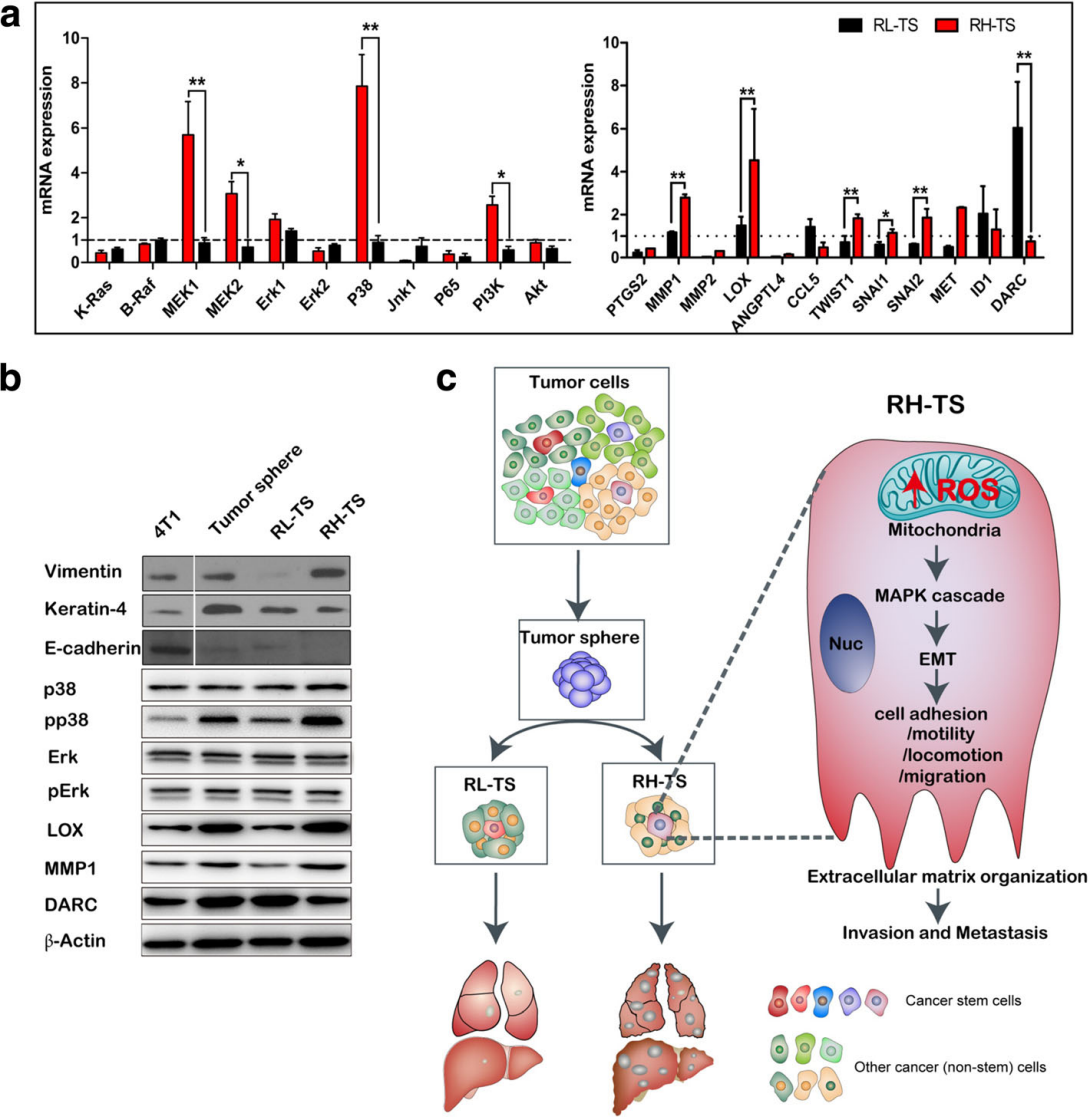
Elevated mitochondrial ROS level promoted the epithelial mesenchymal transition via activation of MAPK signaling pathway. **a** RT-PCR analysis of redox-sensitive signaling pathways, including MEK1, MEK2, P38, and PI3K, and EMT related genes including PTGS2, MMP1, MMP2, LOX, ANGPTL4, CCL5, TWIST1, SNAI1, SNAI2, MET, ID1, and DARC in RH-TS cells and RL-TS cells. Results are shown as mean ± SEM from three independent experiments. * p < 0.05, ** p < 0.01, *** p < 0.001. **b** Analysis of redox-sensitive signaling pathways and EMT related proteins. RH-TS cells had downregulation of an epithelial marker E-cadherin, and upregulation of mesenchymal markers vimentin and keratin-4. Redox-sensitive signaling pathways, including MEK1, MEK2, P38, and PI3K were significantly overexpressed in RH-TS compared to RL-TS cells. **c** Proposed mechanism of the mitochondrial ROS promoted cancer metastasis of RH-TS cells. Elevated ROS of tumor sphere activated the p38 MAPK signaling pathway and promoted EMT process thus enforced the metastases formation

We further analyzed the expression of the related redox-sensitive signaling pathways. It showed that the mRNA expression levels of MEK1, MEK2, P38, and PI3K were significantly overexpressed in RH-TS cells compared to that of RL-TS cells (Fig. 7a). To validate the RNA-seq results, we evaluated the protein expression levels of EMT markers by using Western blot, and it showed downregulation of epithelial marker E-cadherin and upregulation of mesenchymal markers vimentin and keratin-4 in RH-TS cells (Fig. 7b). In addition, increased phosphorylation of p38 was observed in RH-TS cells compared to that in parental cells and RL-TS cells, indicating the activation of MAPK signaling pathway.

TWIST1, TWIST2, SNAI1, and SNAI2 are EMT activators. MMPs can also induce EMT through multiple different signaling pathways [36, 37]. Collectively, our data demonstrated that elevated ROS level, especially the mitochondrial ROS level, activated the p38 MAPK signaling pathway, resulting in the EMT of RH-TS cells, and potentiating caner invasion and metastasis (Fig. 7c).

### Fourteen up-regulated genes in RH-TS correlate to survival in cancer patients

**Fig. 8:**
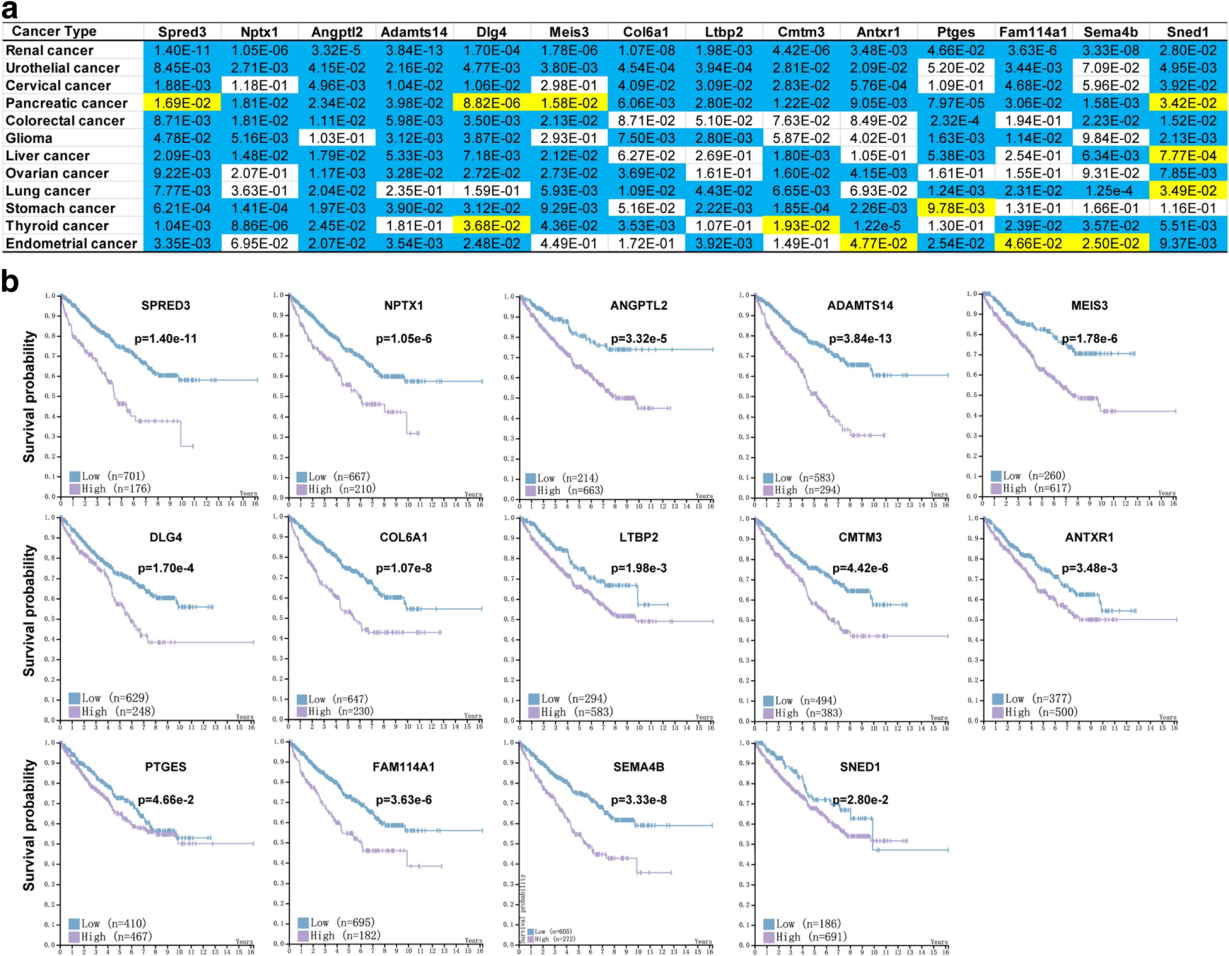
Fourteen up-regulated genes in RH-TS correlated with patient survival negatively. The caner survival analysis of 14 up-regulated genes in RH-TS cells in different cancer patients in the Human Protein Atlas database. **a** Overall survival of patients with renal cancer, urothelial cancer, cervical cancer, pancreatic cancer, colorectal cancer, glioma, liver cancer, ovarian cancer, lung cancer, stomach cancer, thyroid cancer, or endometrial cancer was determined on expression of 14 genes (Spred3, Nptx1, Angptl2, Adamts14, Meis3, Dlg4, Col6a1, Ltbp2, Cmtm3, Antxr1, Ptges, Fam114a1, Sema4b, or Sned1). Color code represents the *P* value for each gene for 5-year survival from different cancer patients. Blue cells indicate that high expression of a certain gene correlated with cancer survival negatively, while yellow cells represent positive correlation. White cells indicate no clear correlation between the gene and cancer survival. **b** The 14 up-regulated genes in RH-TS cells correlated with renal cancer survival negatively

In addition, we examined the relationship between the upregulated genes in RH-TS cells and cancer survival in different cancer patients in the Human Protein Atlas database [38]. As shown in Fig. 8 and Additional file 2: Figure S9-S22, the Kaplan-Meier survival analysis revealed that high expression of 14 upregulated genes (Spred3, Nptx1, Angptl2, Adamts14, Meis3, Dlg4, Col6a1, Ltbp2, Cmtm3, Antxr1, Ptges, Fam114a1, Sema4b, or Sned1) significantly reduced the overall survival in patients with renal cancer, urothelial cancer, cervical cancer, pancreatic cancer, colorectal cancer, glioma, liver cancer, ovarian cancer, lung cancer, stomach cancer, thyroid cancer, and endometrial cancer. Therefore, the elevated mitochondrial ROS level of tumor sphere cells can also be a useful metastatic trait.

## Discussion

Here, we demonstrated that the tumor spheres possessed the intrinsic properties of CSCs and were composed by two heterogeneous populations of cells: RH-TS and RL-TS. The RH-TS cells exhibited elevated mitochondrial ROS level and promoted metastasis formation. RH-TS cells were also characterized by upregulated expression of genes in fatty acid β-oxidation, MAP kinase cascades, and EMT. Moreover, 14 up-regulated genes in RH-TS cells were associated with poor overall survival in different types of cancers.

The redox status specifically in CSCs and its role in cancer metastasis are controversial. Belle et al. demonstrated that high endogenous ROS levels promote the self-renewal and neurogenesis in proliferative neural stem cells [39]. Myant et al. showed that ROS accumulation becomes critical for human colorectal CSCs transformation and tumor initiation [10]. Ishikawa et al. indicated that increased mitochondrial ROS could contribute to tumor progression by enhancing the metastatic potential of tumor cells [40]. However, Ito et al. showed that higher ROS levels limit the lifespan of stem cells such as hematopoietic stem cells [41]. Dong et al. reported that a metabolic switch to glycolysis following loss of fructose-1,6-biphosphatase, which inhibits oxygen consumption and ROS production, is required for the development of basal-like breast cancer [19, 42]. The role of ROS in cancer metastasis remains in great flux, partially because of the heterogeneity of CSCs. Recently, strands of evidence converge on the conclusion that redox homeostasis is the linchpin for CSCs in self-renewal, differentiation, and cancer metastasis. Furthermore, ROS play an important role in determining the fate of stem cells in a concentration-dependent manner. It is known that the intracellular ROS levels oscillate with changes in metabolic activity, and the mitochondria, as the metabolic center of mammalian cells, have been considered to be the major source of cellular-derived ROS because most cellular O_2_ consumption occurs as a result of mitochondrial ETC activity. Thus, the mitochondria might play a central role in ROS-mediated cancer metastasis. Indeed, emerging evidence has demonstrated the pivotal roles of mitochondria and mitochondrial ROS in cellular redox homeostasis and cancer metastasis [43, 44]. Therefore, mitochondrial ROS have been suggested to be novel targets for cancer therapy [45, 46]. Here, we showed that RH-TS cells displayed higher mitochondrial ROS levels and increased cancer metastasis than RL-TS cells. RH-TS cells elevated fatty acid β-oxidation resulting in an increased NADH level, oxygen consumption of mitochondrial ETC, and ROS production. These results suggest that fatty acid β-oxidation may be a therapeutic target, and the underlying mechanisms of broader involvement of fatty acid β-oxidation in RH-TS cells might be a focus of future investigations. Indeed, fatty acid β-oxidation has recently been shown to contribute to mitochondrial spare respiratory capacity and promote the stemness and chemoresistance of breast CSCs [47].

Moreover, it has been established recently that endogenous ROS function as second messengers to stimulate CSCs self-renewal and metastasis via reversibly modulating redox-sensitive pathways. p38 MAPK, possessing a reactive cysteine residue which can be activated by ROS via oxidation, is considered to be redox sensors and works as a mediator of ROS-regulated CSCs metastasis. Bhandari et al. argued that p38 MAPK, as a crucial regulator of redox sensor molecule, controlled the balance between MSC proliferation and differentiation [48]. Invasion and metastasis begins with EMT, and several studies have documented that ROS play the crucial role in EMT through directly interacting with many critical EMT-inducing pathways [49, 50]. ROS production is vital to anoikis resistance for circulating tumor cells via the activation of PI3K/AKT and ERK signaling [51, 52]. Increasing evidence has shown that ROS play a role in creating a ‘soil’ in distal organs, setting up a supportive tumor environment for disseminated cancer cells [53–55]. Our data suggest that mitochondrial ROS might serve as signaling molecules linking fatty acid β-oxidation and EMT phenotype in RH-TS cells, and support the critical role of ROS and EMT in CSCs.

In addition to a role in redox regulation, ROS might also function to alter the epigenetic landscape, which plays a particularly pertinent role in regulating stem cell fate. Many metabolic intermediates are necessary substrates for the post-translational modifications of histones that together establish the epigenetic landscape of stem cells. As the activity of glycolysis and oxidative phosphorylation can directly influence ROS, leading to changes in the concentrations of various metabolic intermediates, this might represent a potential mechanism of ROS-mediated epigenetic regulation, albeit indirect. Given the different cellular oxidation capacities of metastatic CSCs, herein RH-TS, and normal stem cells, perhaps an effective redox targeting therapeutic strategy might be possible to kill metastatic CSCs.

## Conclusions

In conclusion, we identified ROS-high CSCs as metastatic cancer cells, and the elevated mitochondrial ROS might be a useful biomarker of cancer metastasis. Inhibiting the key enzyme of β-oxidation of fatty acids such as CPT1A by pharmacological or genetic manipulations and diminishing cellular mitochondrial ROS level by mitochondrial-specific antioxidant agents such as SkQ1 might be a promising approach to prevent and alleviate cancer metastasis induced by RH-TS cells.

## Additional file

Additional file 1: The ARRIVE Guidelines Checklist S1. Animal Research: Reporting In Vivo Experiments. (DOCX 623 kb)

Additional file 2: Supplementary materials, methods, figures and tables. (DOCX 30583 kb)

## Abbreviations

CSCs: Cancer Stem Cells
DAF-FM: 4-amino-5-methylamino-29,79-difluorofluorescein
DCFH-DA: 2′,7′-Dichlorodihydrofluorescein diacetate
DHE: dihydroethidium
MitoSOX Red: MitoSOX TM Red mitochondrial superoxide indicator
RH-TS: ROS-High Tumor Spheres
RL-TS: ROS-Low Tumor Spheres
ROS: Reactive oxygen species
TICs: tumor-initiating cells
