# Supplementary material for: Elevated level of mitochondrial reactive oxygen species via fatty acid β-oxidation in cancer stem cells promotes cancer metastasis by inducing epithelial–mesenchymal transition
Source: Stem Cell Res Ther. 2019 Jun 13;10:175. doi: 10.1186/s13287-019-1265-2 (PMC6567550; doi:10.1186/s13287-019-1265-2)

**Supplementary materials for**

**Elevated level of mitochondrial reactive oxygen species via fatty acid β-oxidation in cancer stem cells promotes cancer metastasis by inducing epithelial–mesenchymal transition**

Caihua Wang^1*^, Liming Shao^1*^, Chi Pan^2^, Jun Ye^1^, Zonghui Ding^3^, Jia Wu^1^, Qin Du^1^, Yuezhong Ren^4#^, Chunpeng Zhu^1#^

1 Department of Gastroenterology, the Second Affiliated Hospital, ZhejiangUniversity School of Medicine, Hangzhou 310009, China.

2 Department of Surgical Oncology, the Second Affiliated Hospital, Zhejiang University School of Medicine, Hangzhou 310009, China.

3 Department of Biochemistry and Molecular Biology, Mayo Clinic Arizona, Scottsdale, Arizona 85259

4 Department of Endocrinology and Metabolism, the Second Affiliated Hospital, Zhejiang University School of Medicine, Hangzhou 310009, China.

* These authors contributed equally to this work.

# To whom correspondence should be addressed.

ChunpengZhu, Department of Gastroenterology, The Second Affiliated Hospital, ZhejiangUniversity School of Medicine, Hangzhou 310009, China. E-mail: [zhuchunpeng@zju.edu.cn](mailto:zhuchunpeng@zju.edu.cn)

Yuezhong Ren, Department of Endocrinology and Metabolism, The Second Affiliated Hospital, Zhejiang University School of Medicine, Hangzhou310009, China. E-mail: renyuez@zju.edu.cn


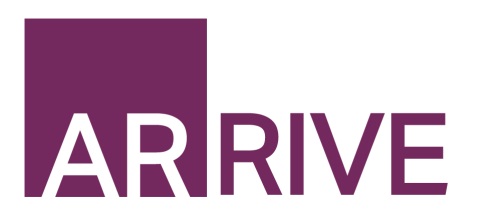


The ARRIVE Guidelines Checklist S1

Animal Research: Reporting In Vivo Experiments

|  | | ITEM | RECOMMENDATION | Section/ Paragraph |
| --- | --- | --- | --- | --- |
| 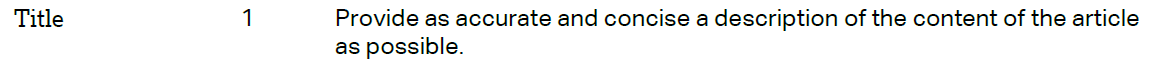 | | | Title |  |
| 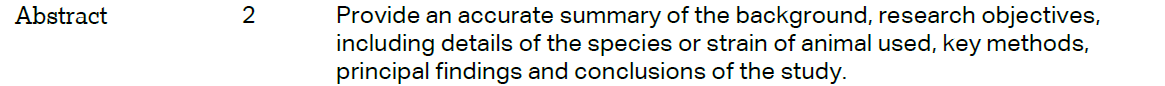 | | | Abstract |  |
| INTRODUCTION | | |  |  |
| 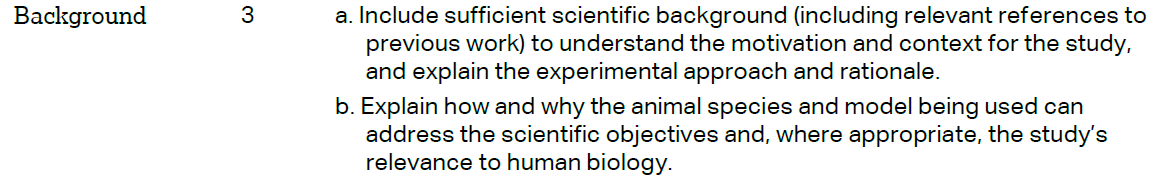 | | | Paragraphs 1-4  Paragraph 4 |  |
| 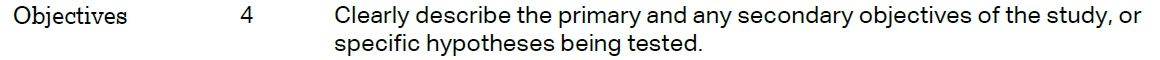 | | | Paragraph 4 |  |
| METHODS | | |  |  |
| 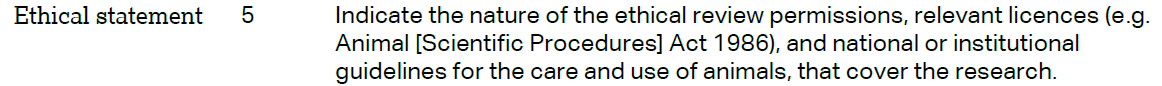 | | | Methods Paragraph 8;  Ethics approval paragraph |  |
| 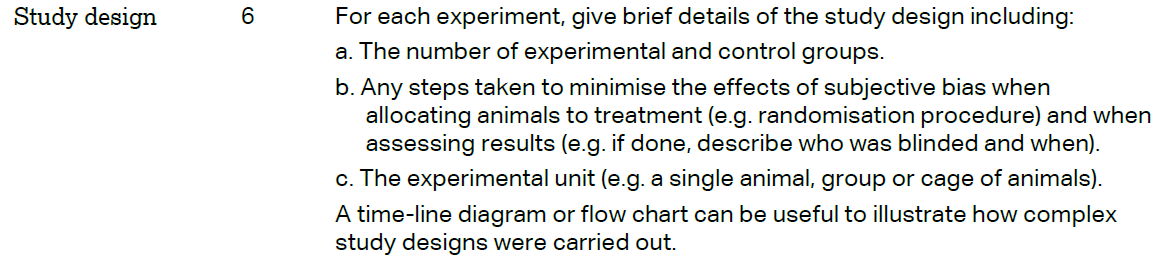 | | | Methods Paragraph 8 -9  Methods Paragraphs 8-9  Methods Paragraphs 8-9 |  |
| 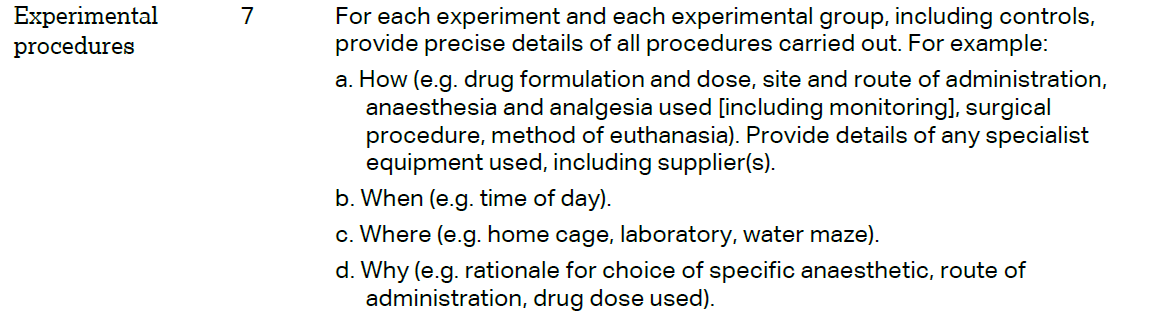 | | | Methods Paragraphs 8-9  Methods Paragraphs 8-9  Methods Paragraphs 8-9 |  |
| 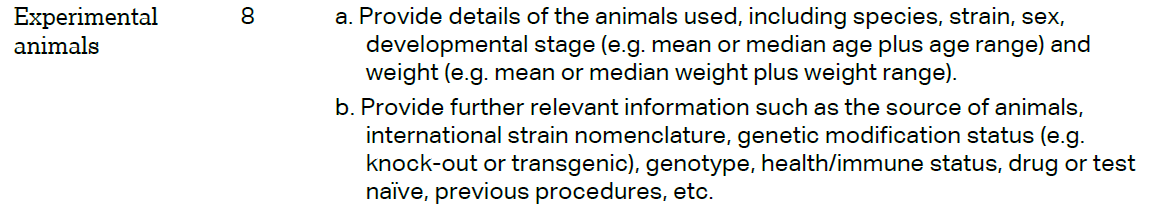 | | | Methods Paragraphs 8-9  Methods Paragraphs 8-9 |  |

| 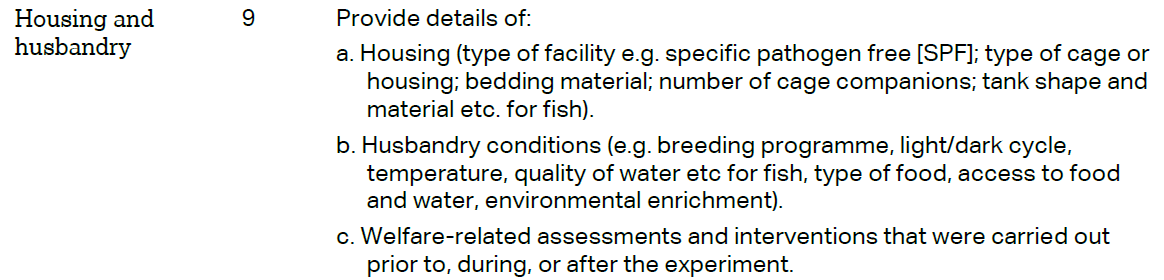 | Methods Paragraphs 8-9  Methods Paragraphs 8-9  Methods Paragraphs 8-9 |  |
| --- | --- | --- |
| 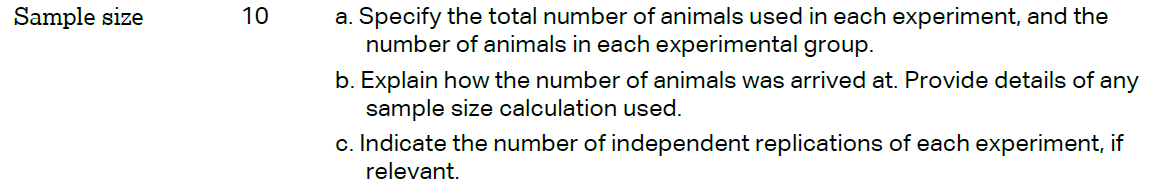 | Methods Paragraphs 8-9  Methods Paragraphs 8-9  Methods Paragraphs 8-9 |  |
| 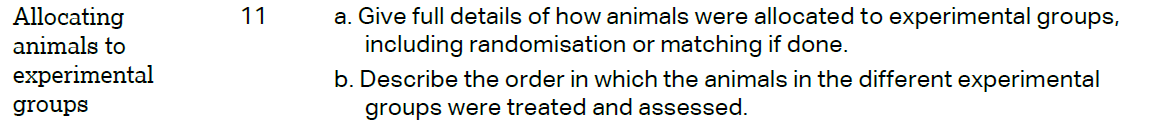 | Methods Paragraphs 9  Methods Paragraphs 9 |  |
| 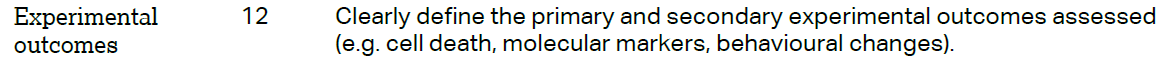 | Methods Paragraphs 9 |  |
| 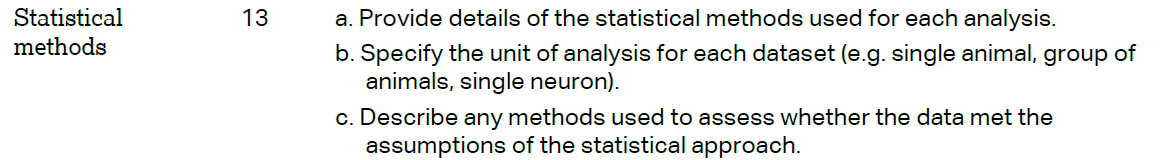 | Methods Paragraphs 16  Methods Paragraphs 16  Methods Paragraphs 16 |  |
| RESULTS |  |  |
| 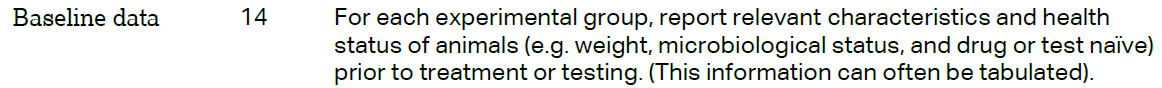 | Results Paragraph 7 |  |
| 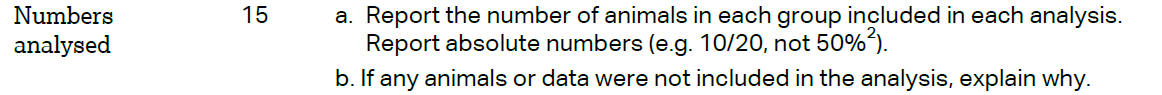 | Results Paragraph 7 |  |
| 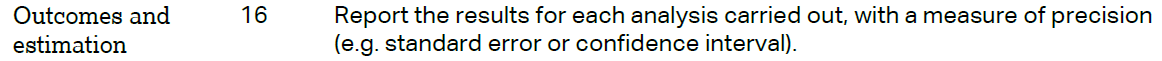 | Results Paragraph 7 |  |
| 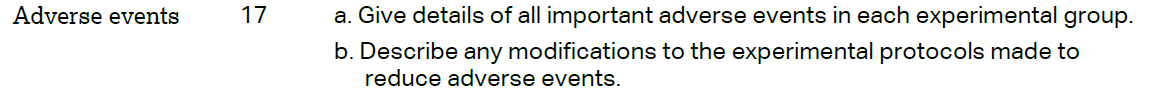 | No  No |  |
| DISCUSSION |  |  |
| 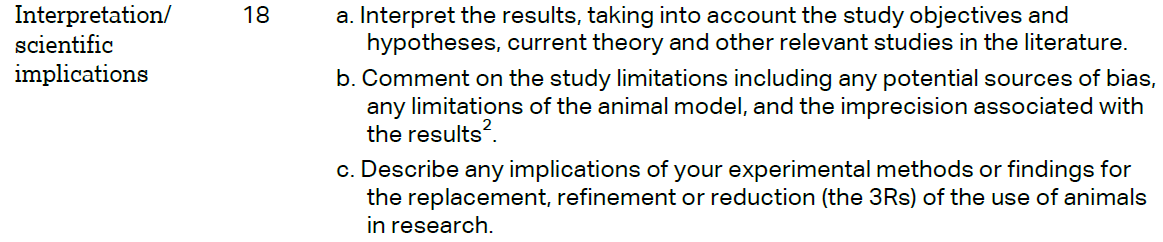 | Throughout  Discussion Paragraph 2  Discussion Paragraph 3-4 |  |
| 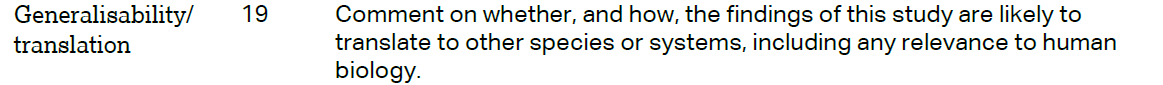 | Conclusions Paragraph |  |
| 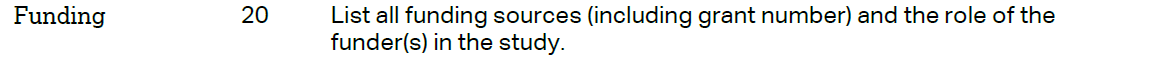 | | Funding Paragraph |


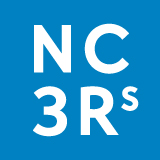

Supplement: Supplementary file 1 — The ARRIVE Guidelines Checklist S1. Animal Research: Reporting In Vivo Experiments. (DOCX 623 kb) [file 13287_2019_1265_MOESM1_ESM.docx]
