# Supplementary material for: Elevated level of mitochondrial reactive oxygen species via fatty acid β-oxidation in cancer stem cells promotes cancer metastasis by inducing epithelial–mesenchymal transition
Source: Stem Cell Res Ther. 2019 Jun 13;10:175. doi: 10.1186/s13287-019-1265-2 (PMC6567550; doi:10.1186/s13287-019-1265-2)
Supplement: Supplementary file 2 — Supplementary materials, methods, figures and tables. (DOCX 30583 kb) [file 13287_2019_1265_MOESM2_ESM.docx]

**Supplementary materials for**

**Elevated level of mitochondrial reactive oxygen species via fatty acid β-oxidation in cancer stem cells promotes cancer metastasis by inducing epithelial–mesenchymal transition**

Caihua Wang^1*^, Liming Shao^1*^, Chi Pan^2^, Jun Ye^1^, Zonghui Ding^3^, Jia Wu^1^, Qin Du^1^, Yuezhong Ren^4#^, Chunpeng Zhu^1#^

1 Department of Gastroenterology, the Second Affiliated Hospital, ZhejiangUniversity School of Medicine, Hangzhou 310009, China.

2 Department of Surgical Oncology, the Second Affiliated Hospital, Zhejiang University School of Medicine, Hangzhou 310009, China.

3 Department of Biochemistry and Molecular Biology, Mayo Clinic Arizona, Scottsdale, Arizona 85259

4 Department of Endocrinology and Metabolism, the Second Affiliated Hospital, Zhejiang University School of Medicine, Hangzhou 310009, China.

* These authors contributed equally to this work.

# To whom correspondence should be addressed.

ChunpengZhu, Department of Gastroenterology, The Second Affiliated Hospital, ZhejiangUniversity School of Medicine, Hangzhou 310009, China. E-mail: [zhuchunpeng@zju.edu.cn](mailto:zhuchunpeng@zju.edu.cn)

Yuezhong Ren, Department of Endocrinology and Metabolism, The Second Affiliated Hospital, Zhejiang University School of Medicine, Hangzhou310009, China. E-mail: renyuez@zju.edu.cn

**Supplementary materials and methods**

**Real-Time PCR**

To validate the gene chip data, we determined the expression of the following genes with real-time PCR (RT-PCR). Total RNA was extracted using RNeasy Mini Kits (Qiagen, USA). The mRNA was reverse transcribed into cDNA using the PrimeScript™ RT Master Mix (RR036A, TaKaRa). Then, 20 ng of cDNA was subjected to quantitative RT-PCR analyses targeting PTGS2, MMP1, MMP2, LOX, ANGPTL4, CCL5, TWIST1, SNAI1, SNAI2, MET, ID1, DARC, K-Ras, B-Raf, MEK1, MEK2, Erk1, Erk2, P38, Jnk1, P65, PI3K and Akt using the SYBR Premix Ex TaqTM (TaKaRa, China). The primer sequences designed with Primer Express software (Applied Biosystems) were listed in Table S3. Analysis was performed using the Step One Real-Time PCR System (Applied Biosystems, Germany). Relative mRNA levels were determined by using the comparative CT (threshold cycle) method. Data was presented as the fold difference in the investigated genes expression normalized to gene β-actin or RPLP0 as endogenous reference, relative to parental 4T1 cells.

**Western blot analysis**

After specific treatment, cells were incubated in lysis buffer (M-PER™ Mammalian Protein Extraction Reagent, 78501, Thermo Fisher Scientific) containing protease inhibitor cocktail (78429, Thermo Fisher Scientific) at 4℃ for 30 minutes. Cell lysates were collected and determined for protein content. Proteins were resolved under denaturing conditions by 10% SDS-PAGE and transferred into a nitrocellulose membrane. The membranes were blocked for 1 hour in 5% nonfat dry milk in TBST (25mM Tris-HCl, pH 7.4, 125mM sodium chloride, 0.05% Tween 20) and incubated with appropriate primary antibodies (Anti-E Cadherin antibody ab133597, Abcam; Anti-Vimentin antibody ab45939, Abcam; Anti-Cytokeratin 4 antibody ab200669, Abcam; Anti-P38 antibody D190810, Beyotime biotechnology; Anti-phospho-P38 antibody D155120, Beyotime biotechnology; Anti-Erk antibody D160317, Beyotime biotechnology; Anti-phospho-Erk antibody D155116, Beyotime biotechnology; Anti-LOX antibody D260069, Beyotime biotechnology; Anti-MMP1 antibody ab137332, Abcam; and Anti-DARC antibody ab137044, Abcam) at 4℃ for 10 hours. Membranes were washed twice with TBST for 15min and incubated with HRP-labeled isotype-specific secondary antibodies for 1 hour at room temperature, and then visualized by Western Lighting Plus ECL kit (Perkin Elmer, USA).

**Transwell invasion assay**

Transwell assay were conducted with invasion chamber (Corning Costar, Cambridge, MA) coated with 40 µl Matrigel mixture (BD Biosciences, matrigel: serum-free culture medium =1:7) under the instructions of manufacturer. 4T1 cells (1x10^5^) were incubated with or without H_2_O_2_ (0.1mM) for indicated time at 37°C. The cells were stained with crystal violet, and observed under a phase-contrast microscope (Carl Zeiss, Axiovert-S100, Germany). The extents of invasion were analyzed by Image J software.

**Supplementary Figure s1**


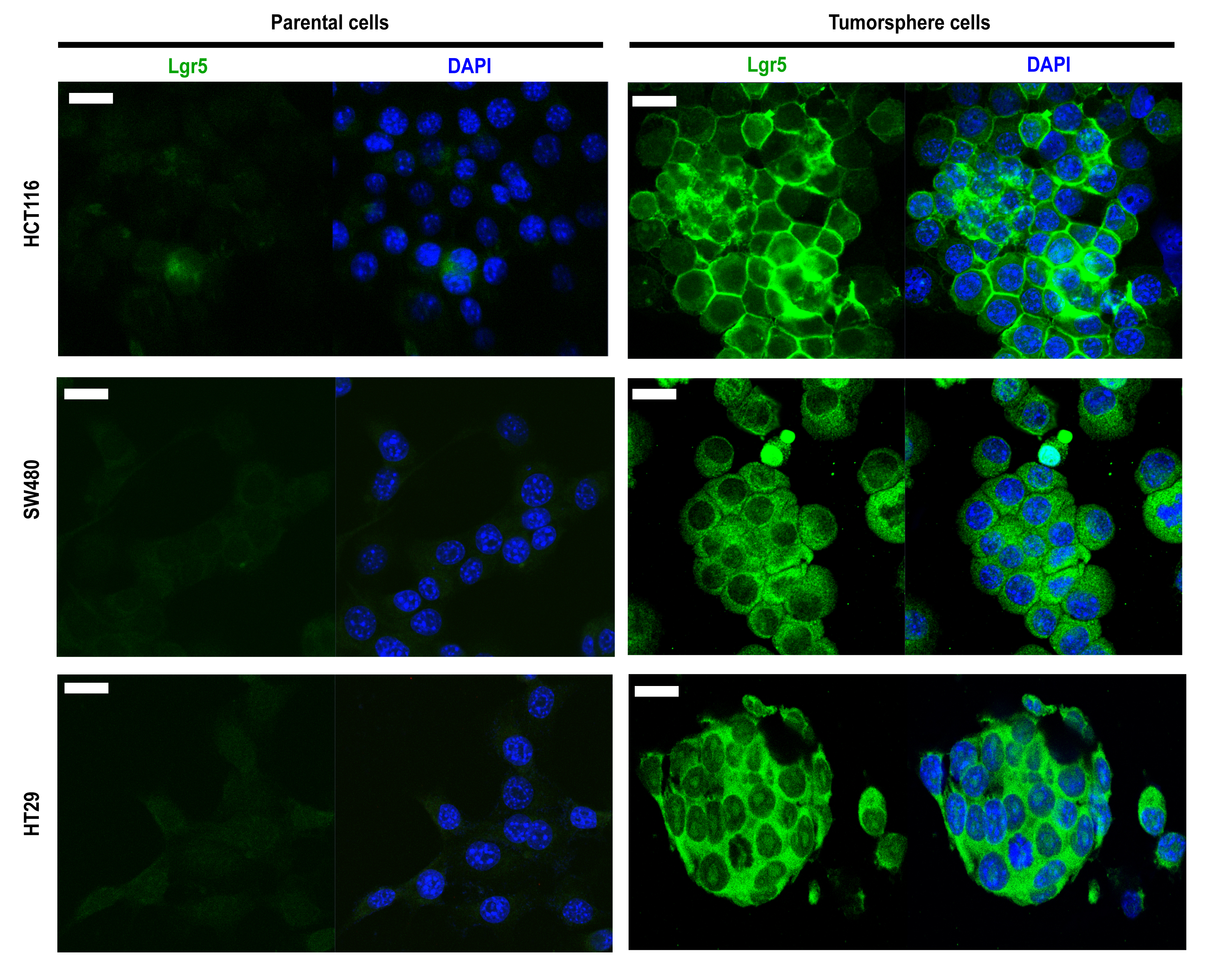


**Fig. S1. Colon cancer tumor sphere cells had the features of CSCs.** Immunofluorescent staining of Lgr5 in colon cancer tumorsphere cells. Tumor sphere isolated from HCT116, SW480, HT29 cells were fixed and stained with anti-Lgr5 antibody (ab75732, Abcam, 1/100). Lgr5 was visualized with Alexa Fluor® 488-conjugated goat anti-rabbit antibody (*green*). Cells were counterstained with DAPI solution (*blue*). Scale bars represent 100 μm.

**Supplementary Figure s2**


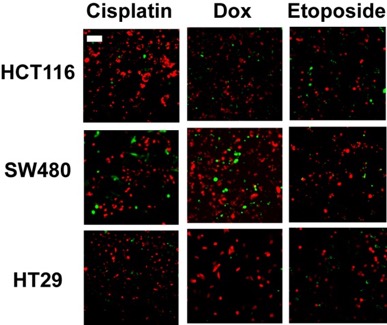


**Fig. S2. Tumor spheres were obviously more resistant to anticancer drugs, such as cisplatin, doxorubicin (Dox), and etoposide than parental cells.** Co-culture of tumor sphere cells (red fluorescence) and parental cells (green fluorescence) with drugs including cisplatin, doxorubicin (Dox), or etoposide. After 2 days incubation, much more tumor sphere cells survived than parental cells. Results represented of three independent experiments. Scale bars represent 100 μm.

**Supplementary Figure s3**

**
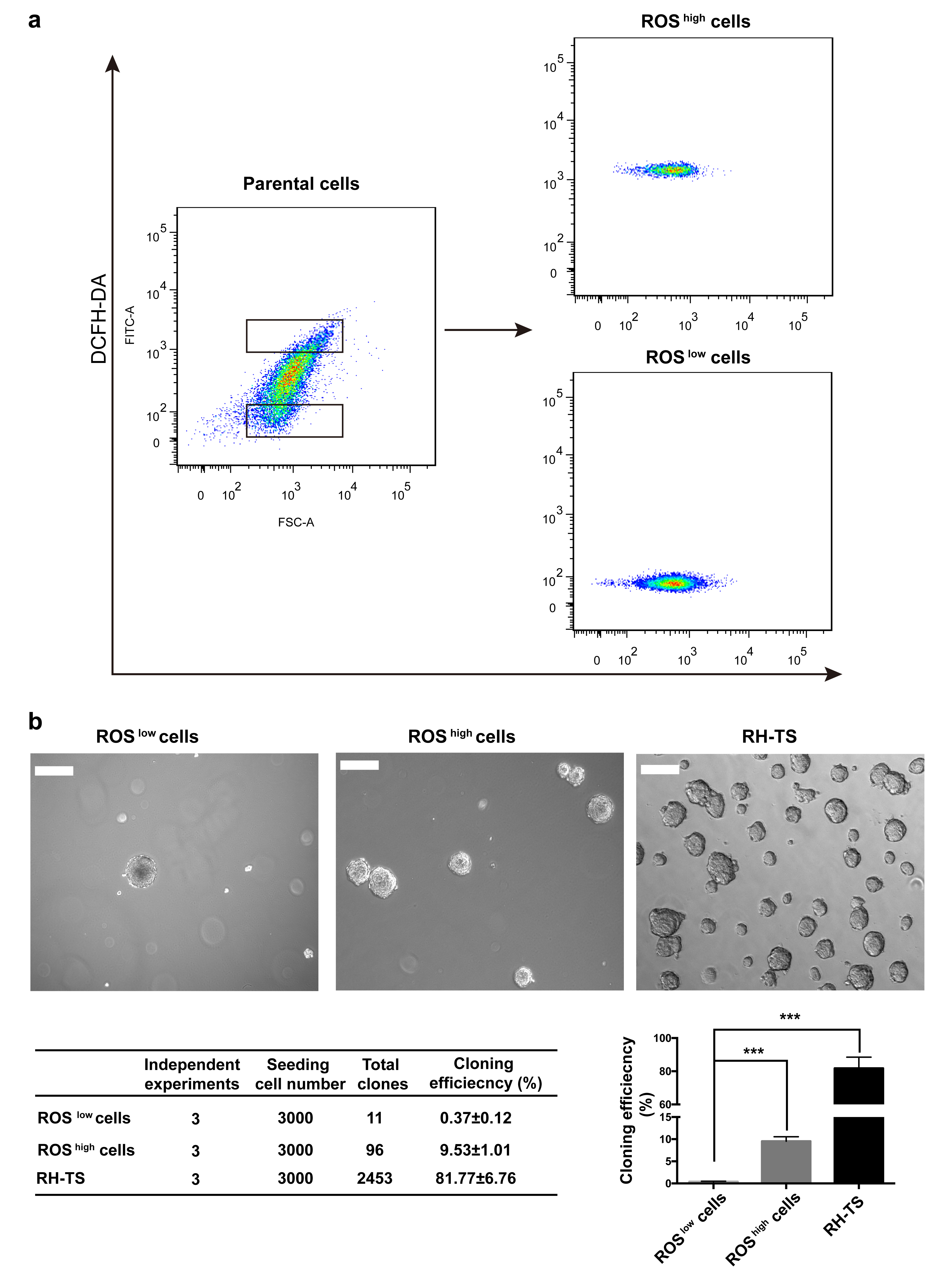
**

**Fig. S3. ROS^high^ cells isolated from parental cells had the features of CSCs.** (a) Sorted out ROS^high^ cells with the same ROS level represented by DCFH-DA as RH-TS cells from 4T1 parental cells by FCM. (b) ROS^high^ cells produced significantly more tumor sphere clones than ROS^low^ cells, but significantly less tumor sphere clones than RH-TS cells. ***P＜0.001. Three independent experiments performed.

**Supplementary Figure s4**

**
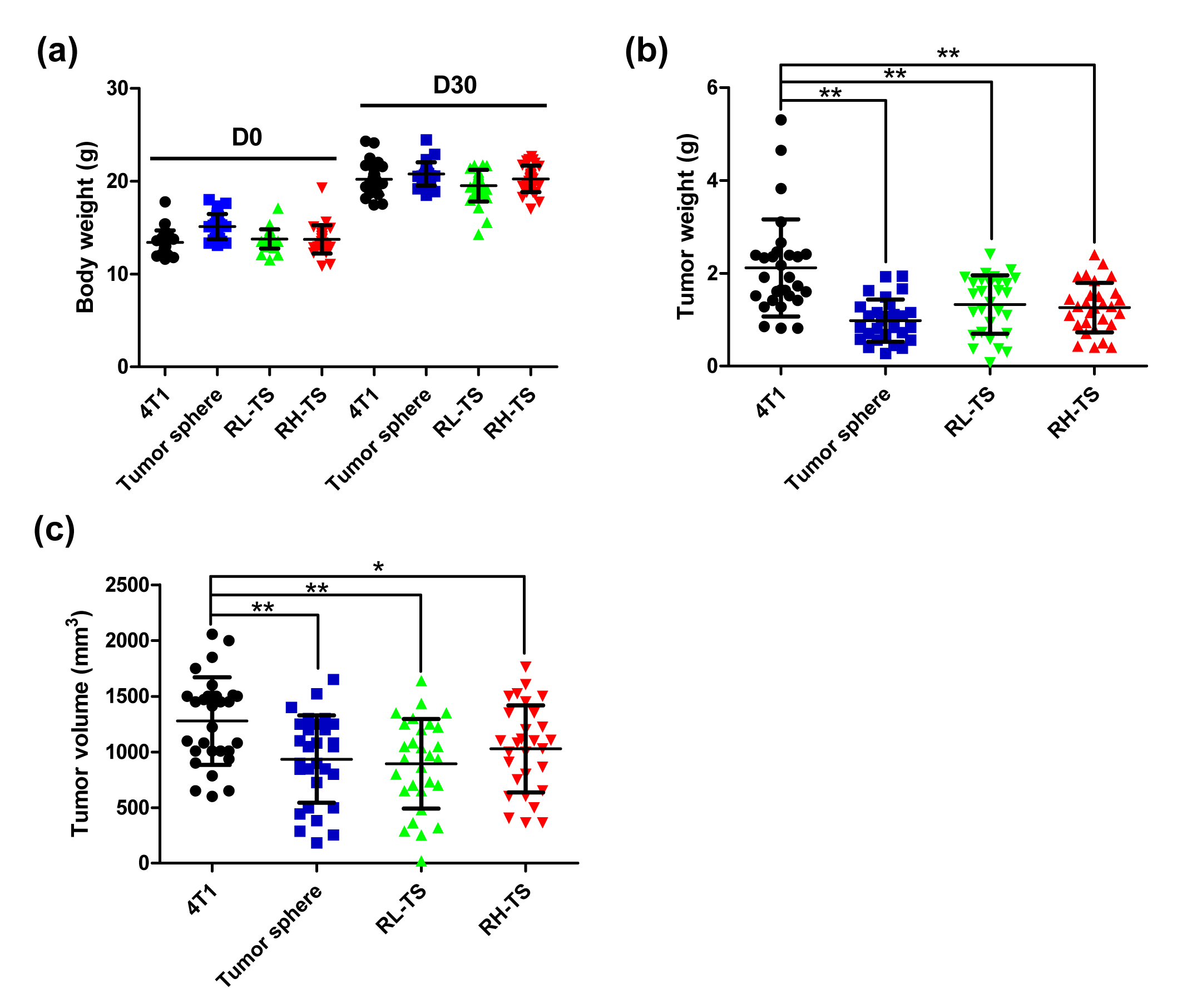
**

**Fig. S4. ROS high-tumor spheres were the principal metastatic source of 4T1 cells.** Tumor spheres, RH-TS, and RL-TS from 4T1 cells were freshly prepared as described in Experimental Procedures. (a) Body weight of BALB/c mice on the day of sacrifice. (b) Tumor weight of BALB/c mice on the day of sacrifice. (c) Tumor size of BALB/c mice on the day of sacrifice. **P＜0.01, *P＜0.05. Five independent experiments performed.

**Supplementary Figure s5**

**
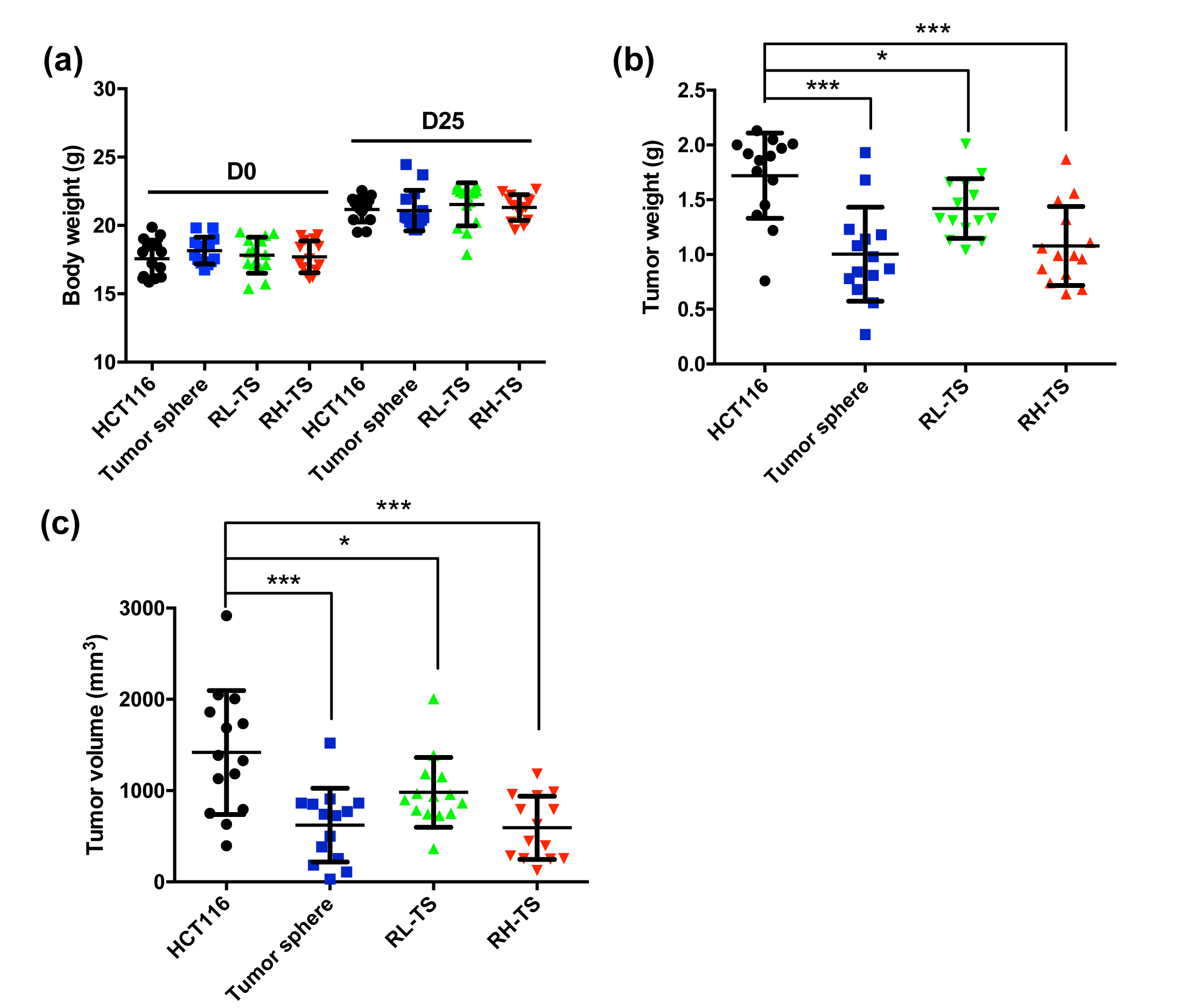
**

**Fig. S5. ROS high-tumor spheres were the principal metastatic source of HCT116 cells.** Tumor spheres, RH-TS, and RL-TS from HCT116 cells were freshly prepared as described in Experimental Procedures. (a) Body weight of NOD/SCID mice on the day of sacrifice. (b) Tumor weight of NOD/SCID mice on the day of sacrifice. (c) Tumor size of NOD/SCID mice on the day of sacrifice. **P＜0.01, *P＜0.05. Two independent experiments performed.

**Supplementary Figure s6**

**
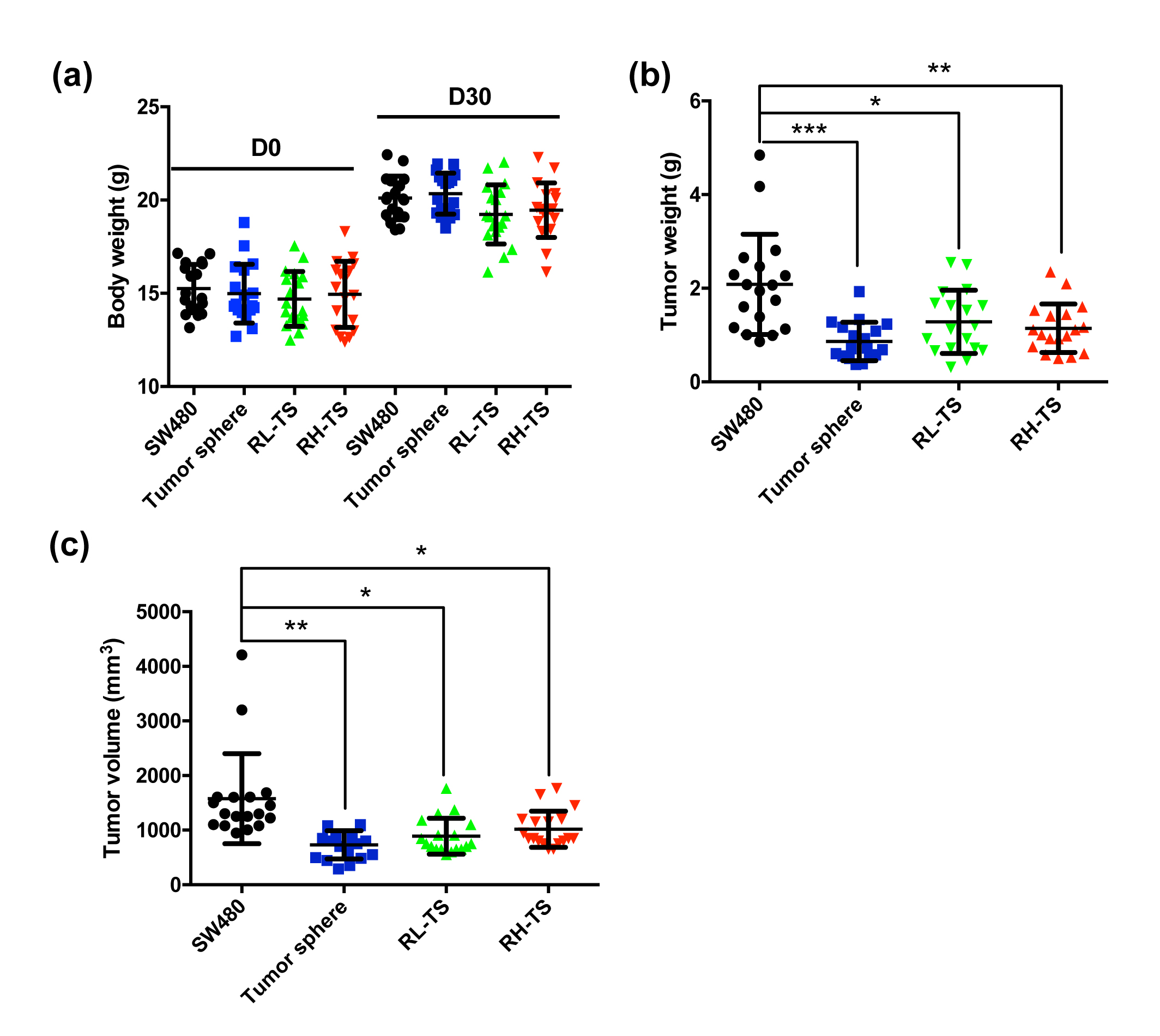
**

**Fig. S6. ROS high-tumor spheres were the principal metastatic source of SW480 cells.** Tumor spheres, RH-TS, and RL-TS from SW480 cells were freshly prepared as described in Experimental Procedures. (a) Body weight of NOD/SCID mice on the day of sacrifice. (b) Tumor weight of NOD/SCID mice on the day of sacrifice. (c) Tumor size of NOD/SCID mice on the day of sacrifice. **P＜0.01, *P＜0.05. Two independent experiments performed.

**Supplementary Figure s7**

**
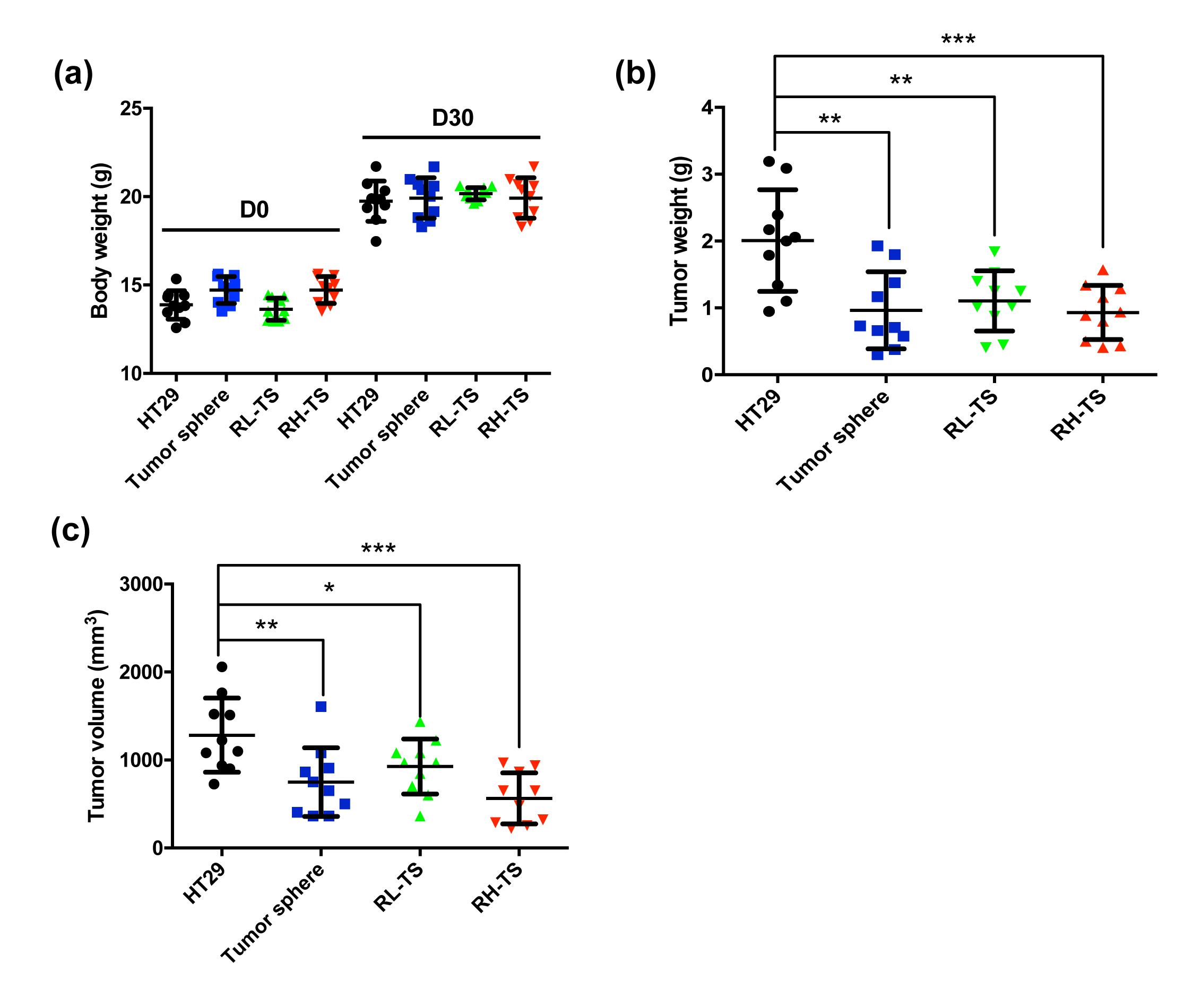
**

**Fig. S7. ROS high-tumor spheres were the principal metastatic source of HT29 cells.** Tumor spheres, RH-TS, and RL-TS from HT29 cells were freshly prepared as described in Experimental Procedures. (a) Body weight of NOD/SCID mice on the day of sacrifice. (b) Tumor weight of NOD/SCID mice on the day of sacrifice. (c) Tumor size of NOD/SCID mice on the day of sacrifice. **P＜0.01, *P＜0.05. Two independent experiments performed.

**Supplementary Figure s8**


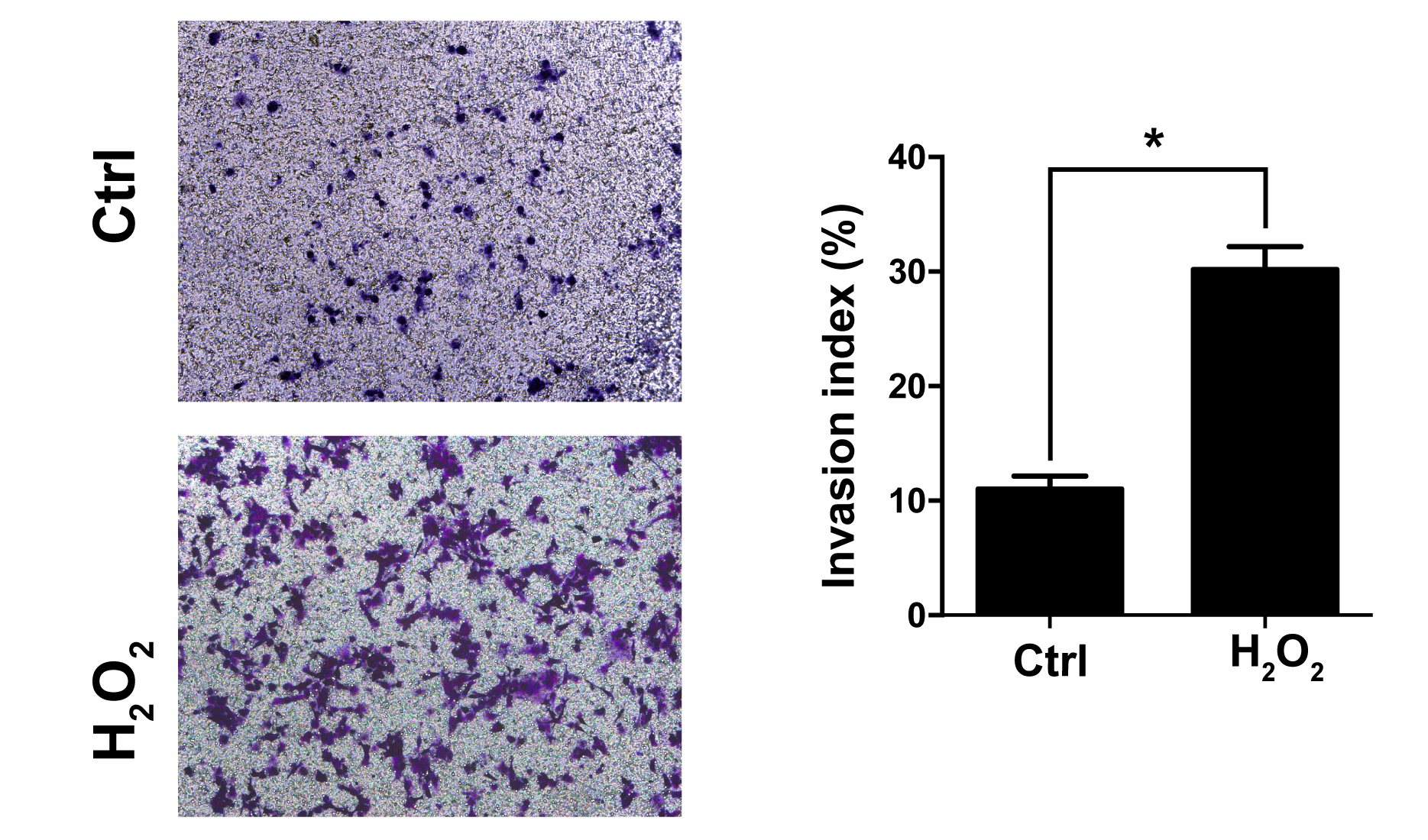


**Fig. S8. ROS could promote cancer cell invasion.** Transwell-invasion assays was used to analyse the invasion ability of 4T1 cells incubated with or without 0.1mM H_2_O_2_. 4T1 cells with H_2_O_2_ incubation exhibited higher invasion ability. *P＜0.05.

**Supplementary Figure s9**

**
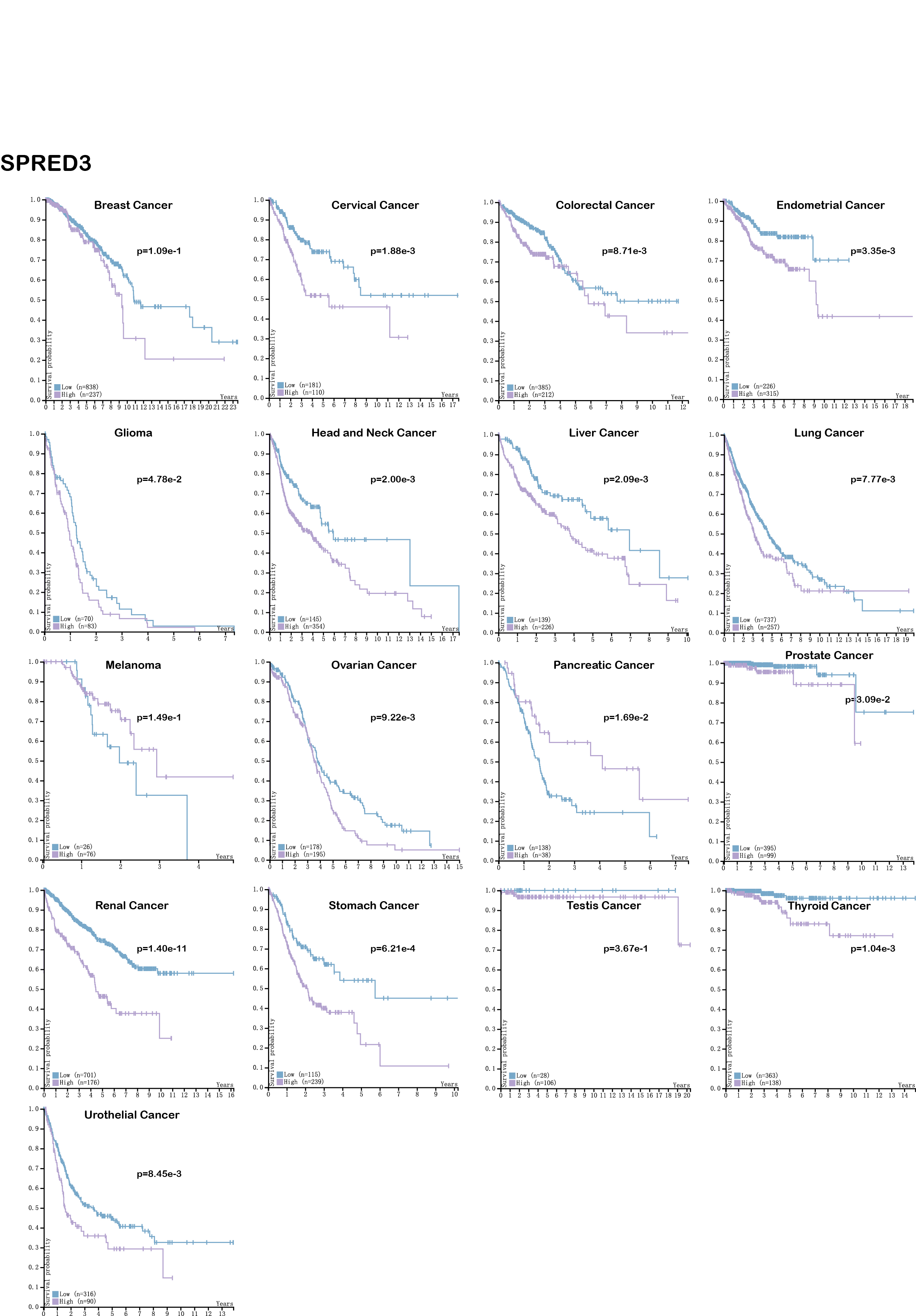
**

**Fig. S9.** Survival analysis of SPRED3 in different cancer patients in the Human Protein Atlas database (http://www.proteinatlas.org).

**Supplementary Figure s10**


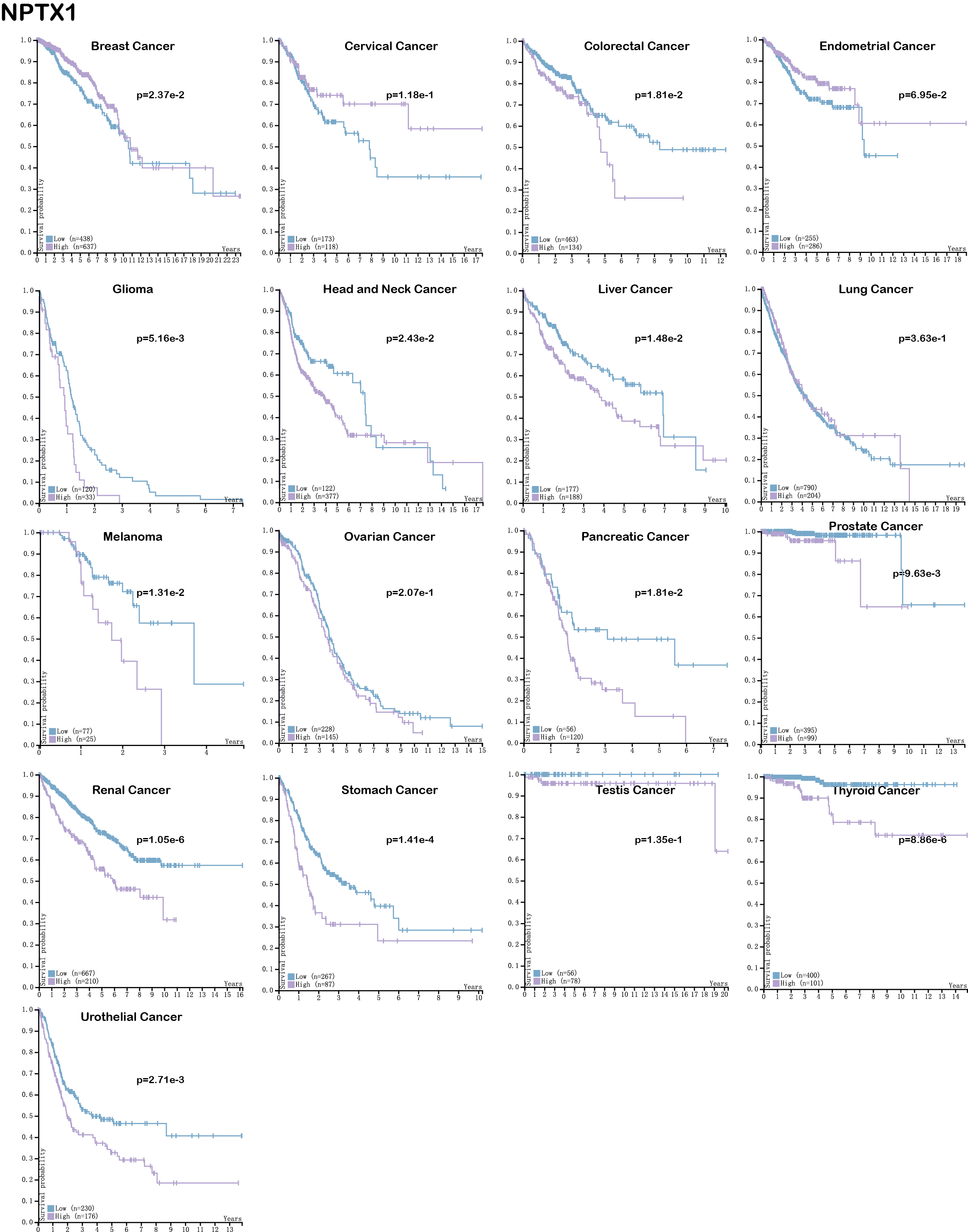


**Fig. S10.** Survival analysis of NPTX1 in different cancer patients in the Human Protein Atlas database (http://www.proteinatlas.org).

**Supplementary Figure s11**


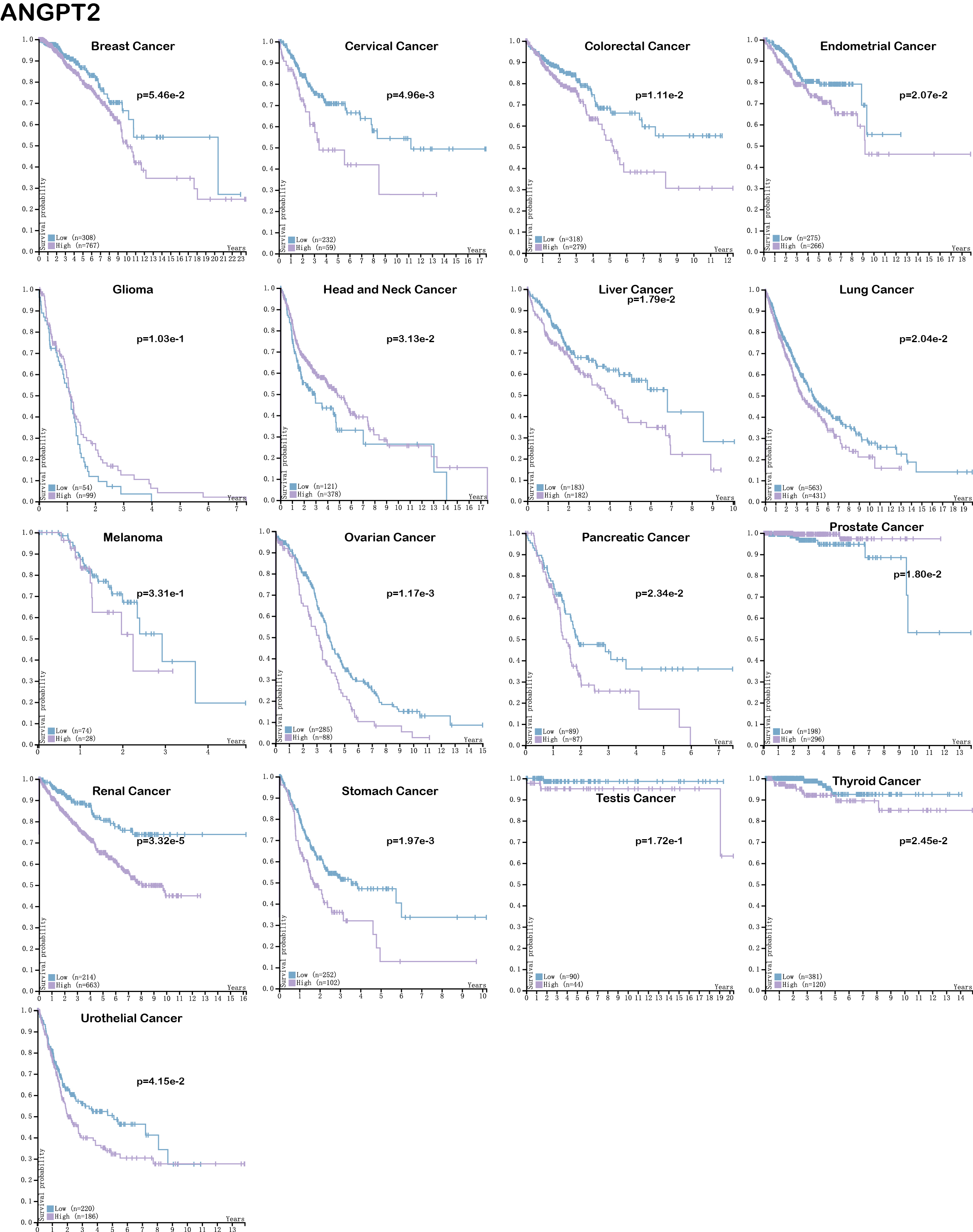


**Fig. S11.** Survival analysis of ANGPT2 in different cancer patients in the Human Protein Atlas database (http://www.proteinatlas.org).

**Supplementary Figure s12**


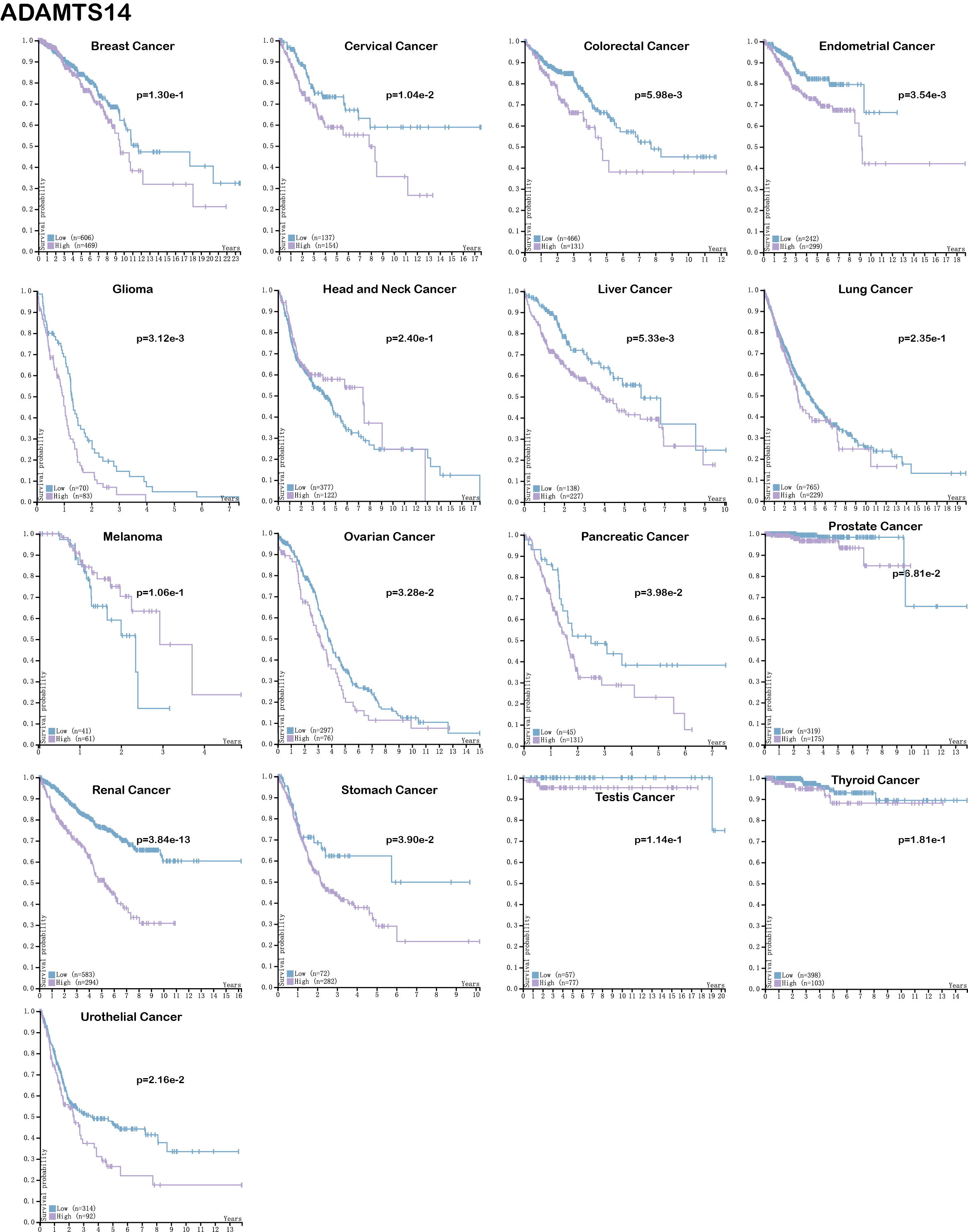


**Fig. S12.** Survival analysis of ADAMTS14 in different cancer patients in the Human Protein Atlas database (http://www.proteinatlas.org).

**Supplementary Figure s13**


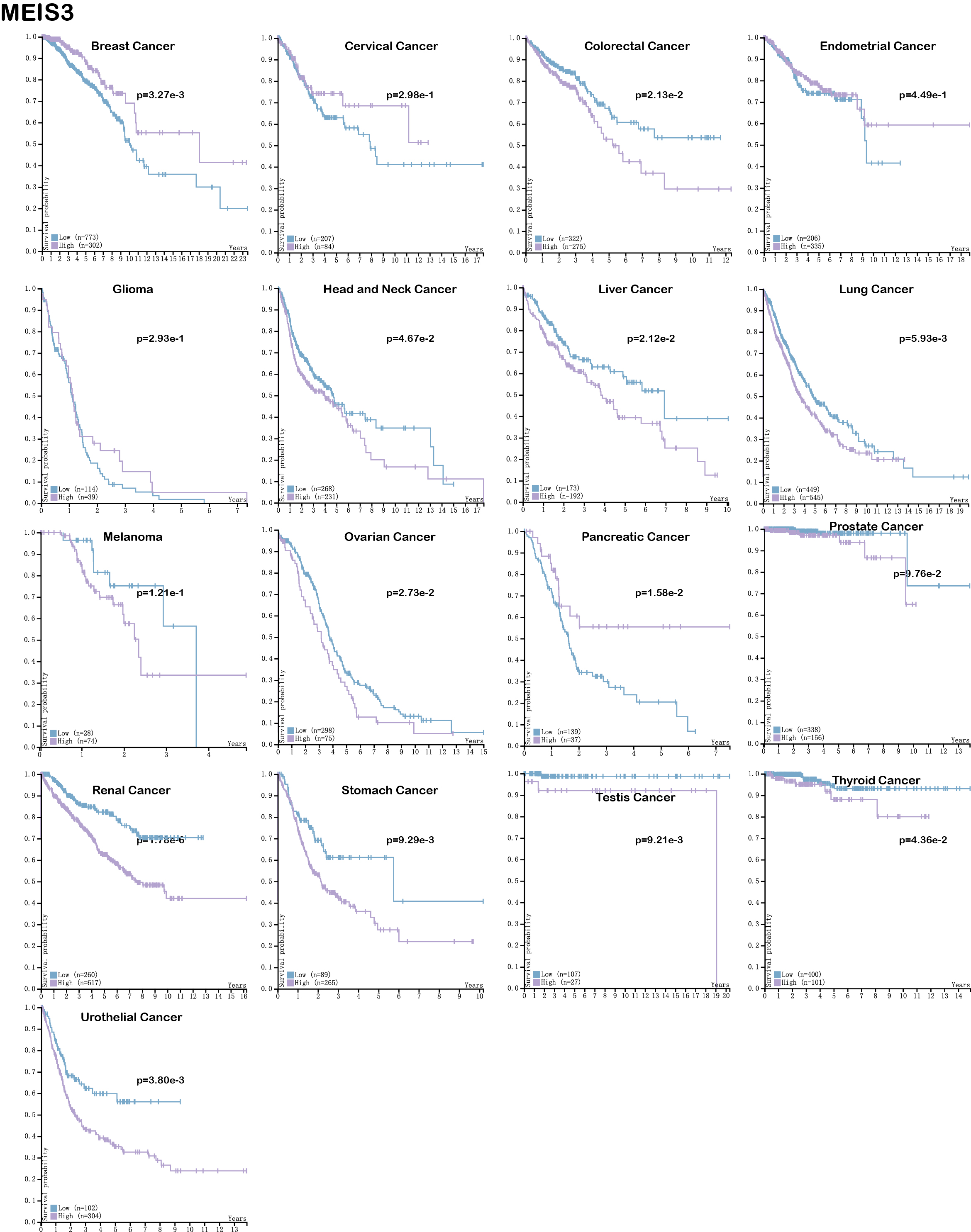


**Fig. S13.** Survival analysis of MEIS3 in different cancer patients in the Human Protein Atlas database (http://www.proteinatlas.org).

**Supplementary Figure s14**


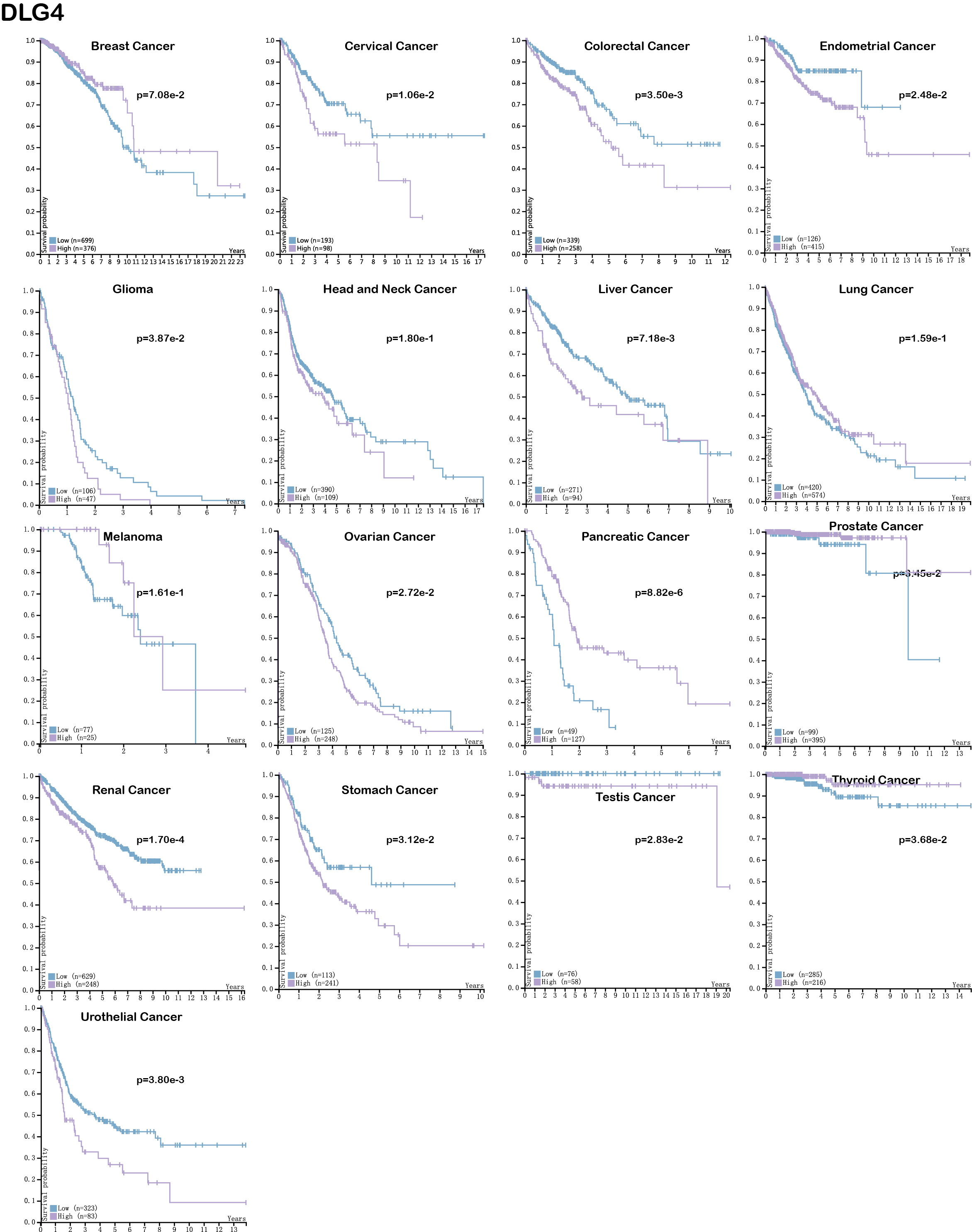


**Fig. S14.** Survival analysis of DLG4 in different cancer patients in the Human Protein Atlas database (http://www.proteinatlas.org).

**Supplementary Figure s15**

**
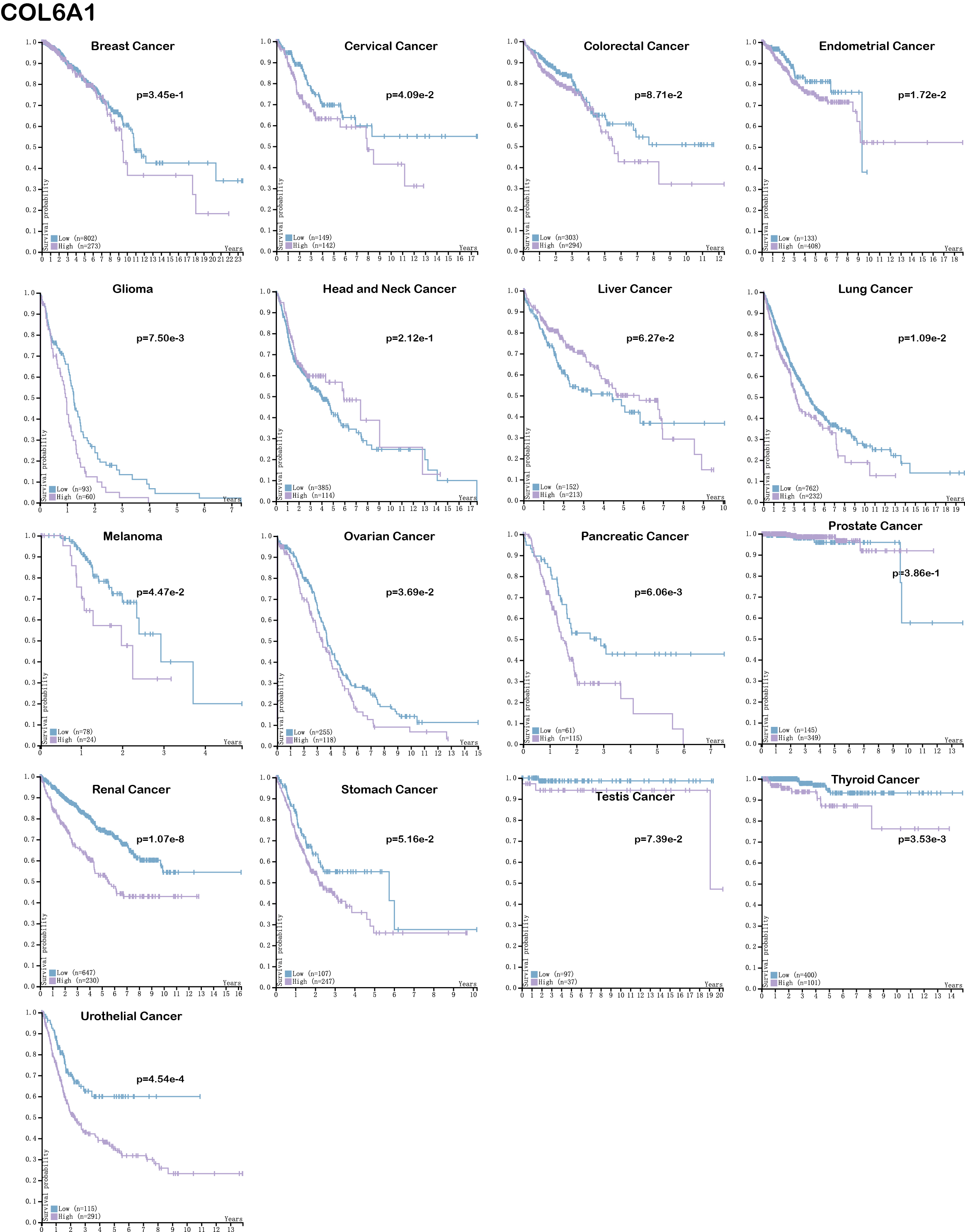
**

**Fig. S15.** Survival analysis of COL6A1 in different cancer patients in the Human Protein Atlas database (http://www.proteinatlas.org).

**Supplementary Figure s16**

**
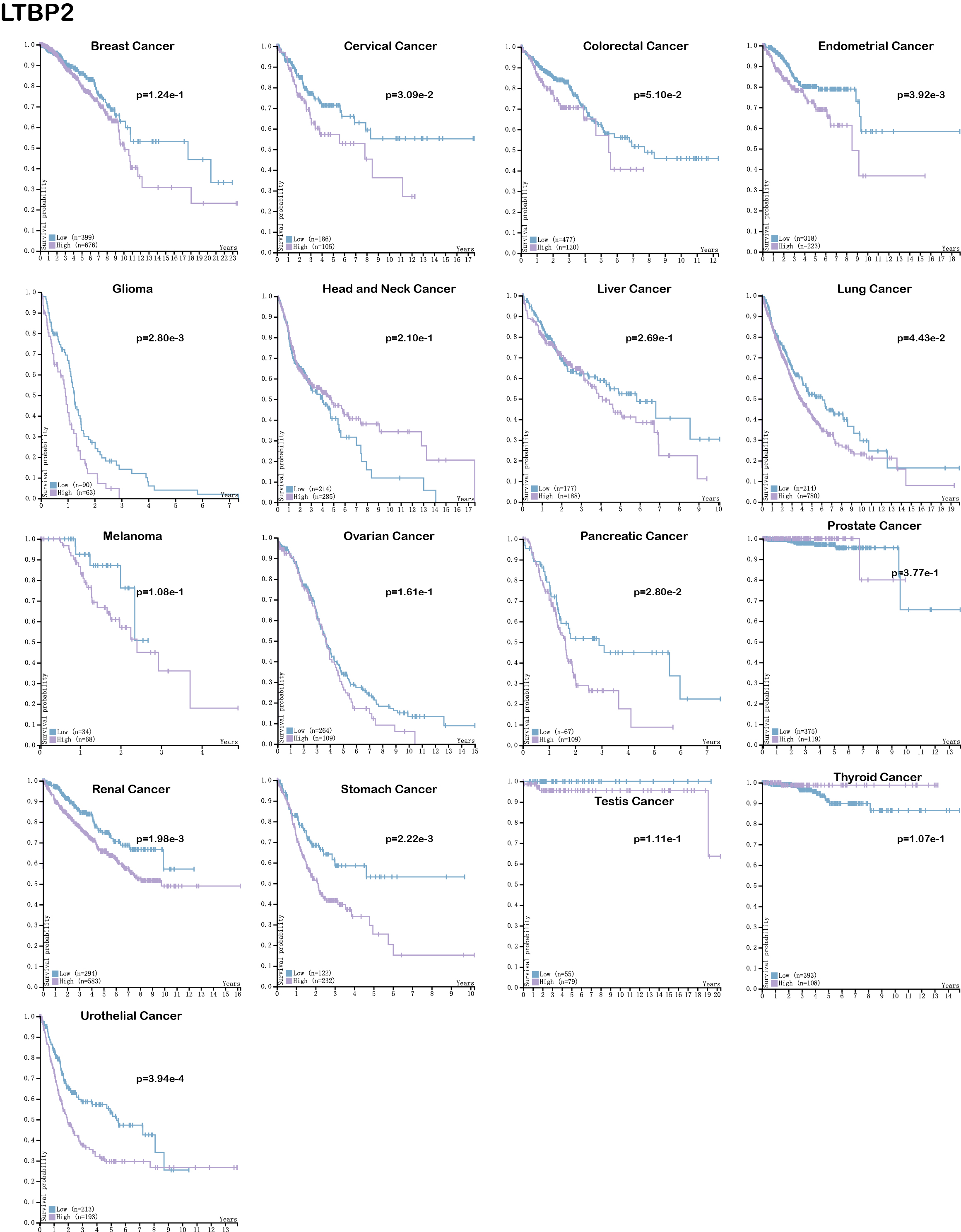
**

**Fig. S16.** Survival analysis of LTBP2 in different cancer patients in the Human Protein Atlas database (http://www.proteinatlas.org).

**Supplementary Figure s17**

**
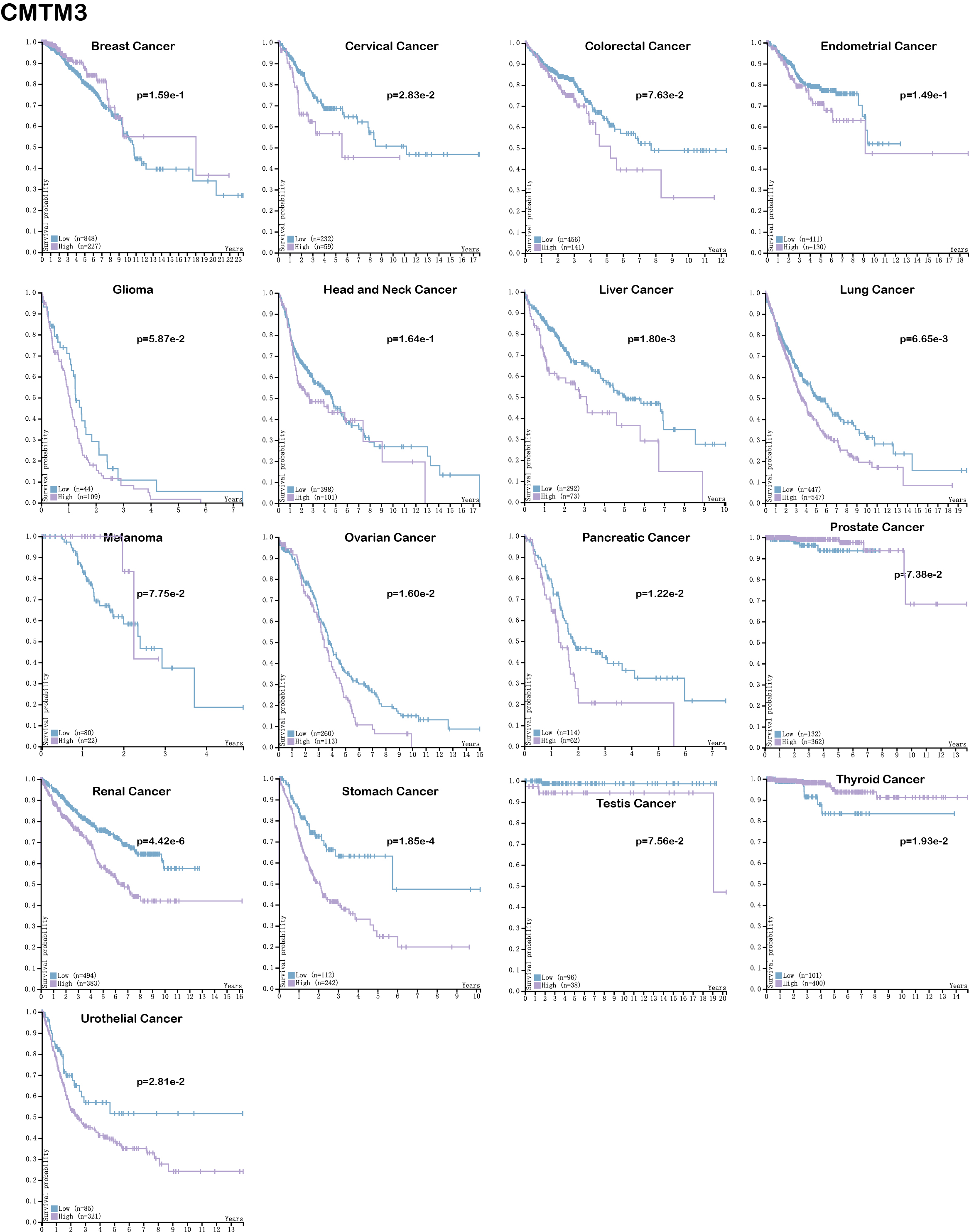
**

**Fig. S17.** Survival analysis of CMTM3 in different cancer patients in the Human Protein Atlas database (http://www.proteinatlas.org).

**Supplementary Figure s18**

**
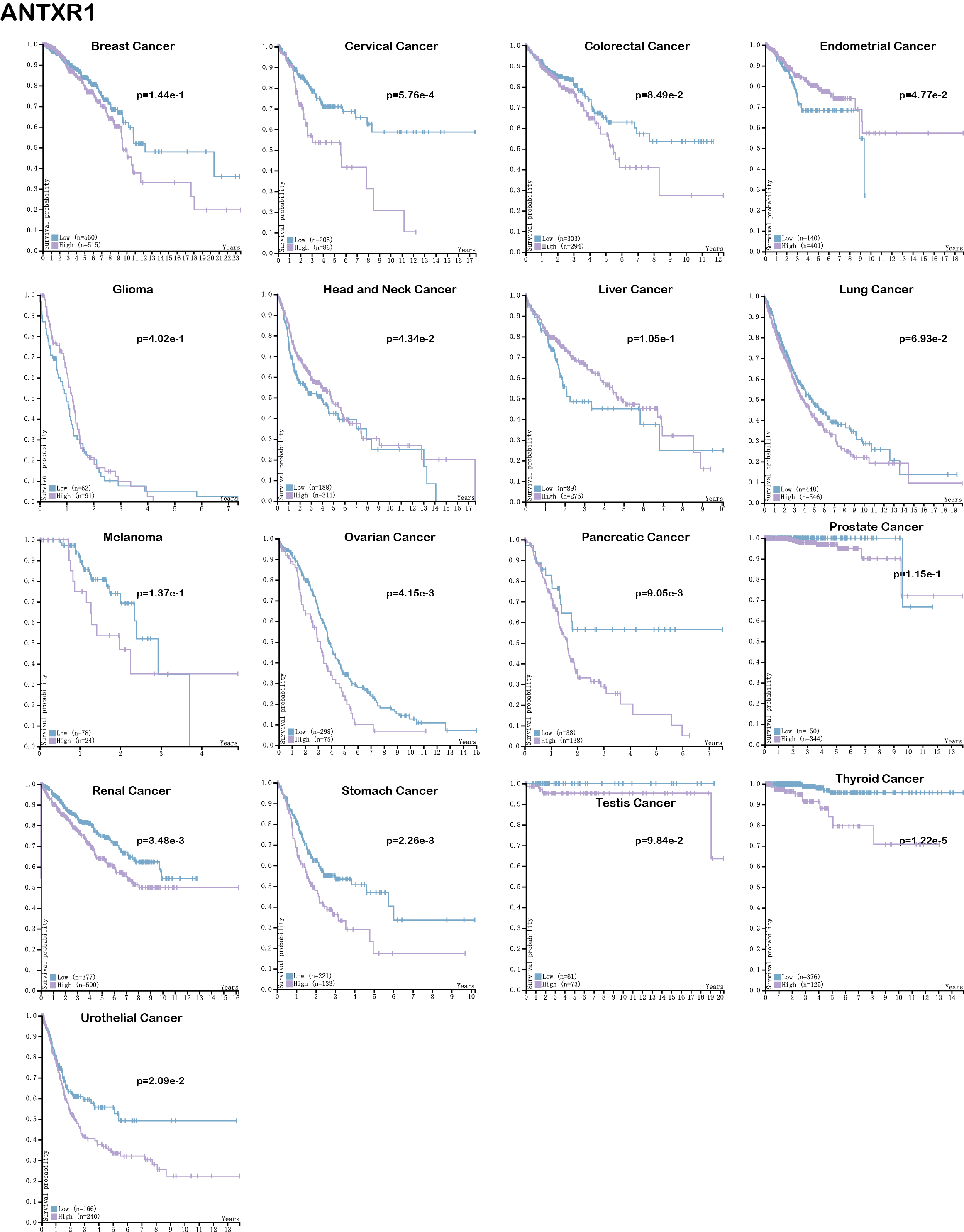
**

**Fig. S18.** Survival analysis of ANTXR1 in different cancer patients in the Human Protein Atlas database (http://www.proteinatlas.org).

**Supplementary Figure s19**

**
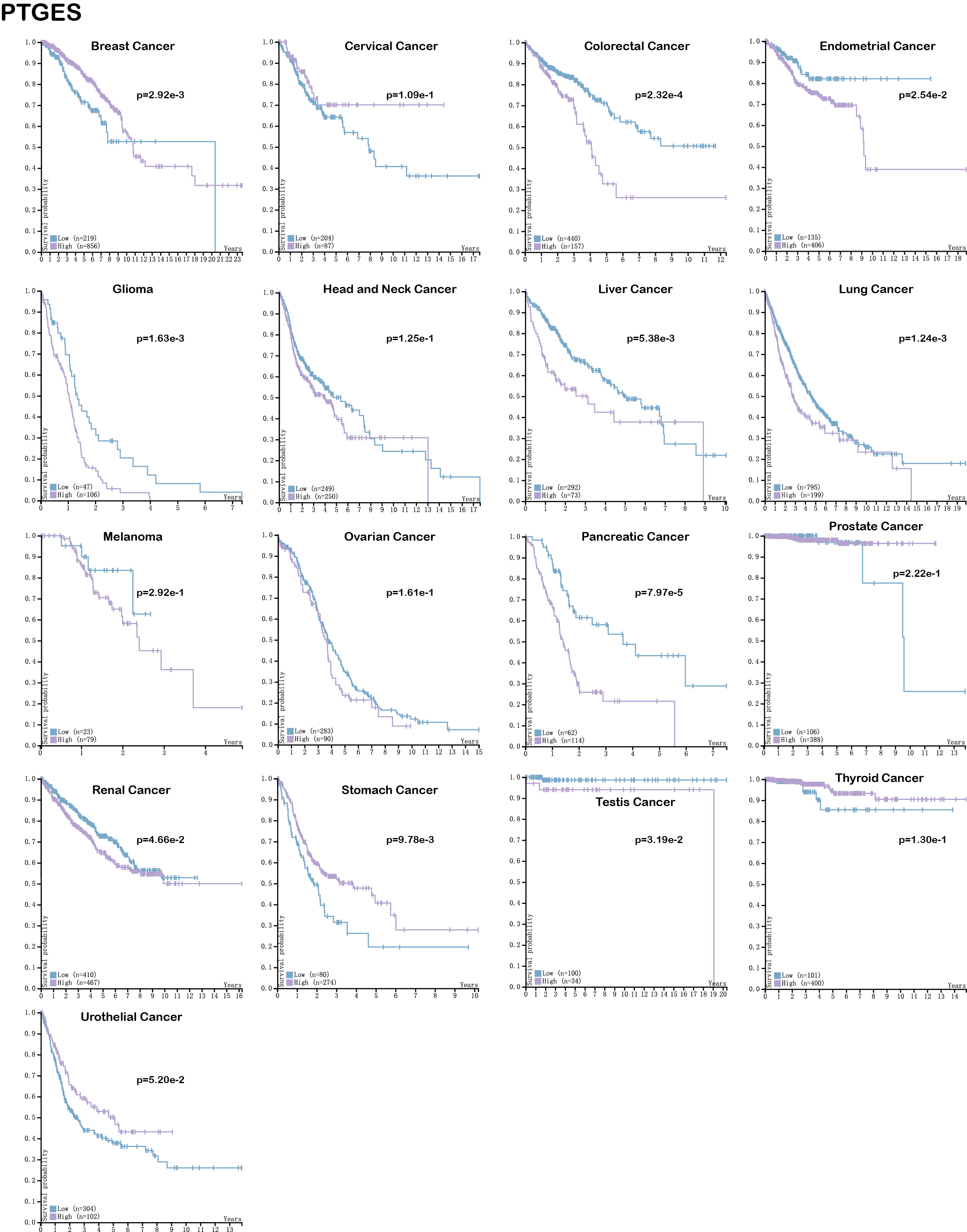
**

**Fig. S19.** Survival analysis of PTGES in different cancer patients in the Human Protein Atlas database (http://www.proteinatlas.org).

**Supplementary Figure s20**

**
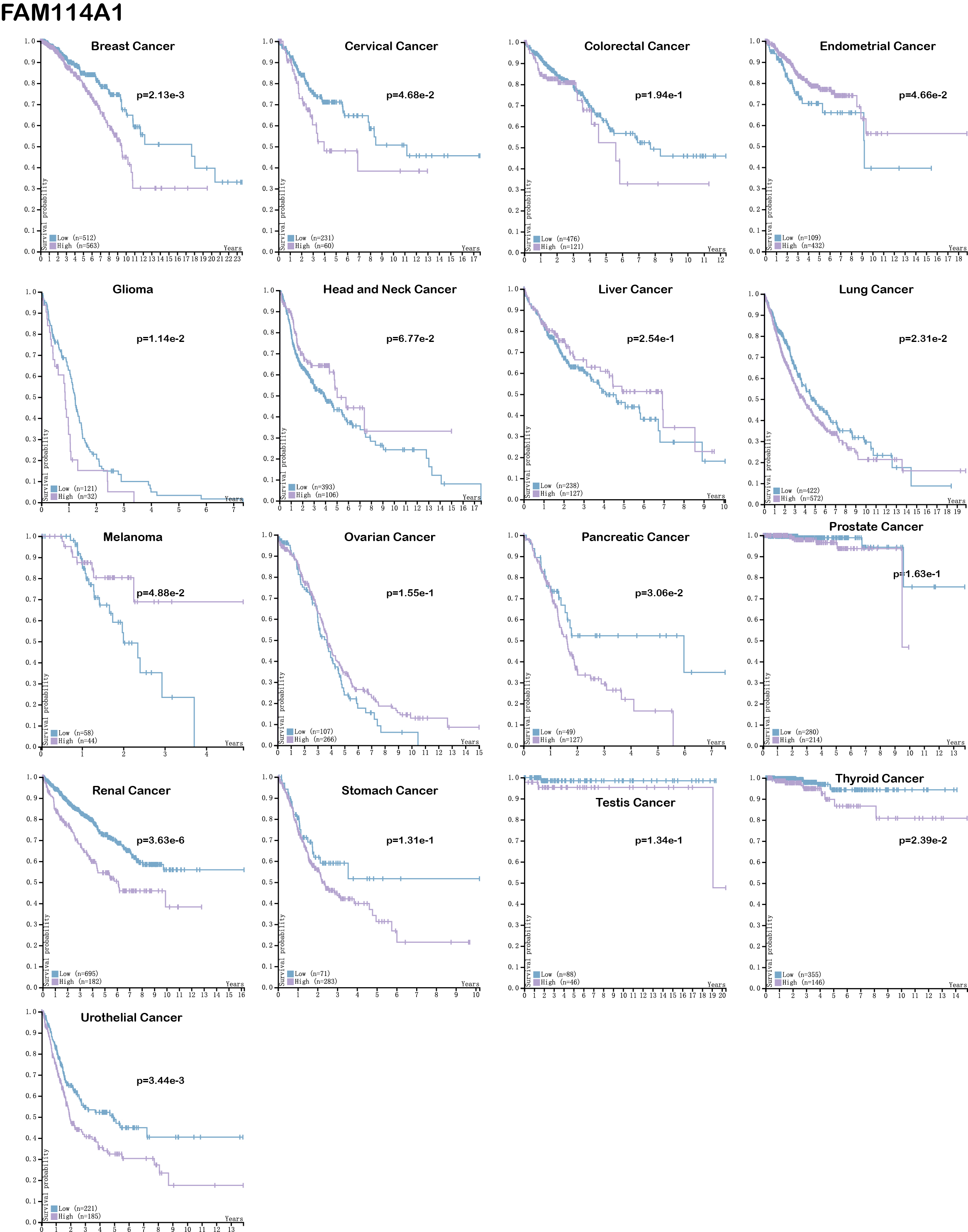
**

**Fig. S20.** Survival analysis of FAM114A1 in different cancer patients in the Human Protein Atlas database (http://www.proteinatlas.org).

**Supplementary Figure s21**

**
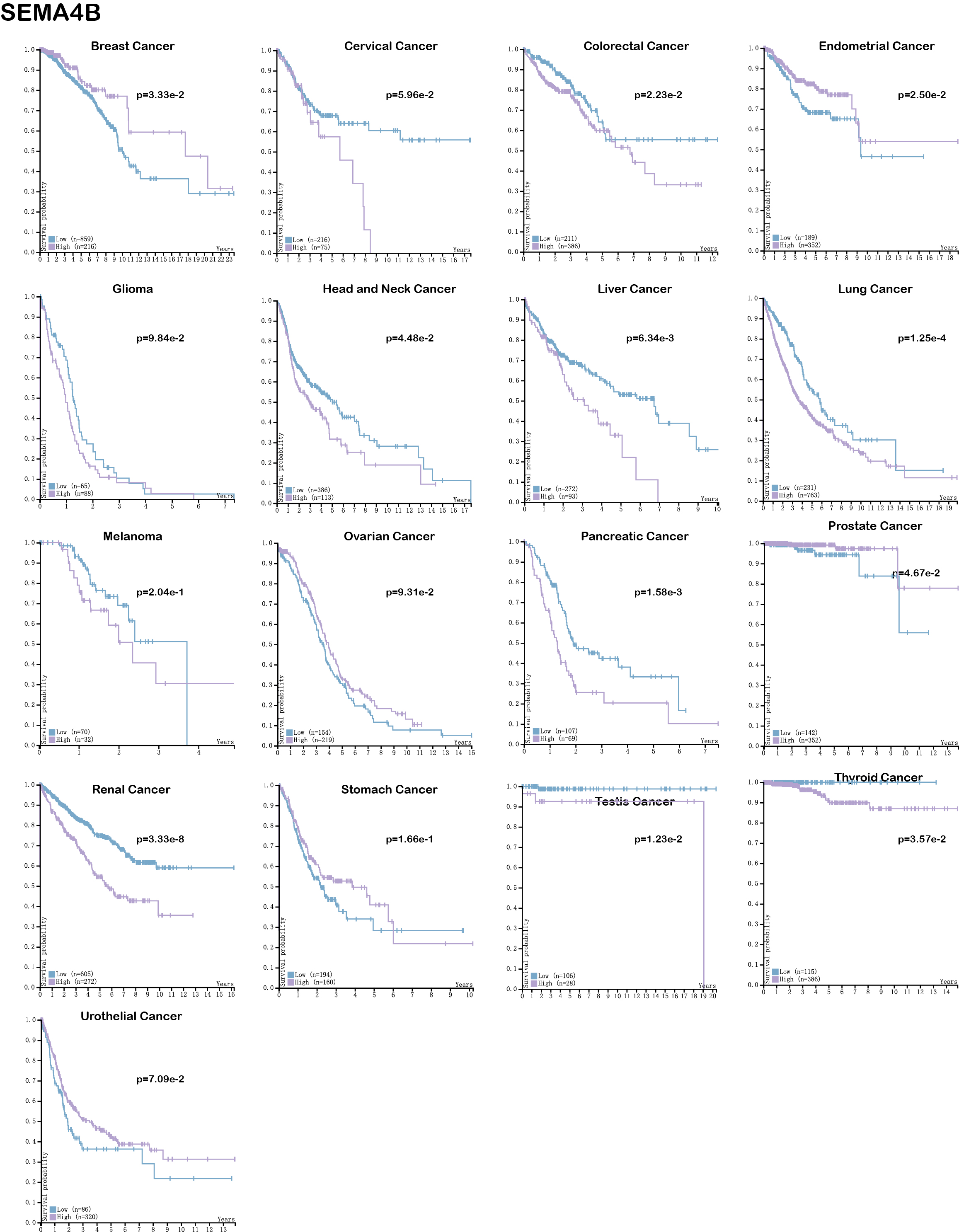
**

**Fig. S21.** Survival analysis of SEMA4B in different cancer patients in the Human Protein Atlas database (http://www.proteinatlas.org).

**Supplementary Figure s22**

**
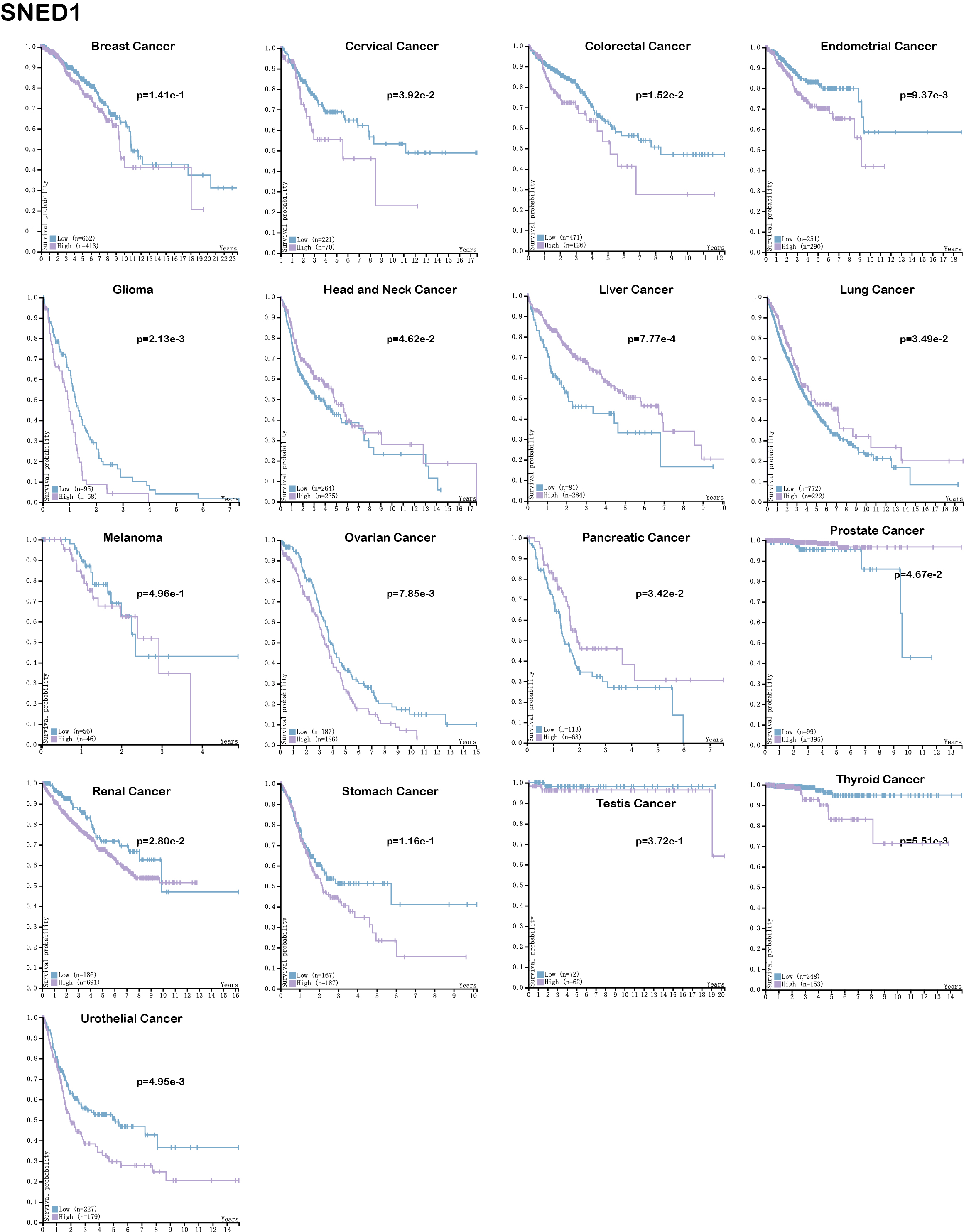
**

**Fig. S22.** Survival analysis of SNED1 in different cancer patients in the Human Protein Atlas database (http://www.proteinatlas.org).

**Supplemental Table s1.** List of 53 genes up-regulated in RH-TS cells as compared to parental cells and RL-TS cells.

**
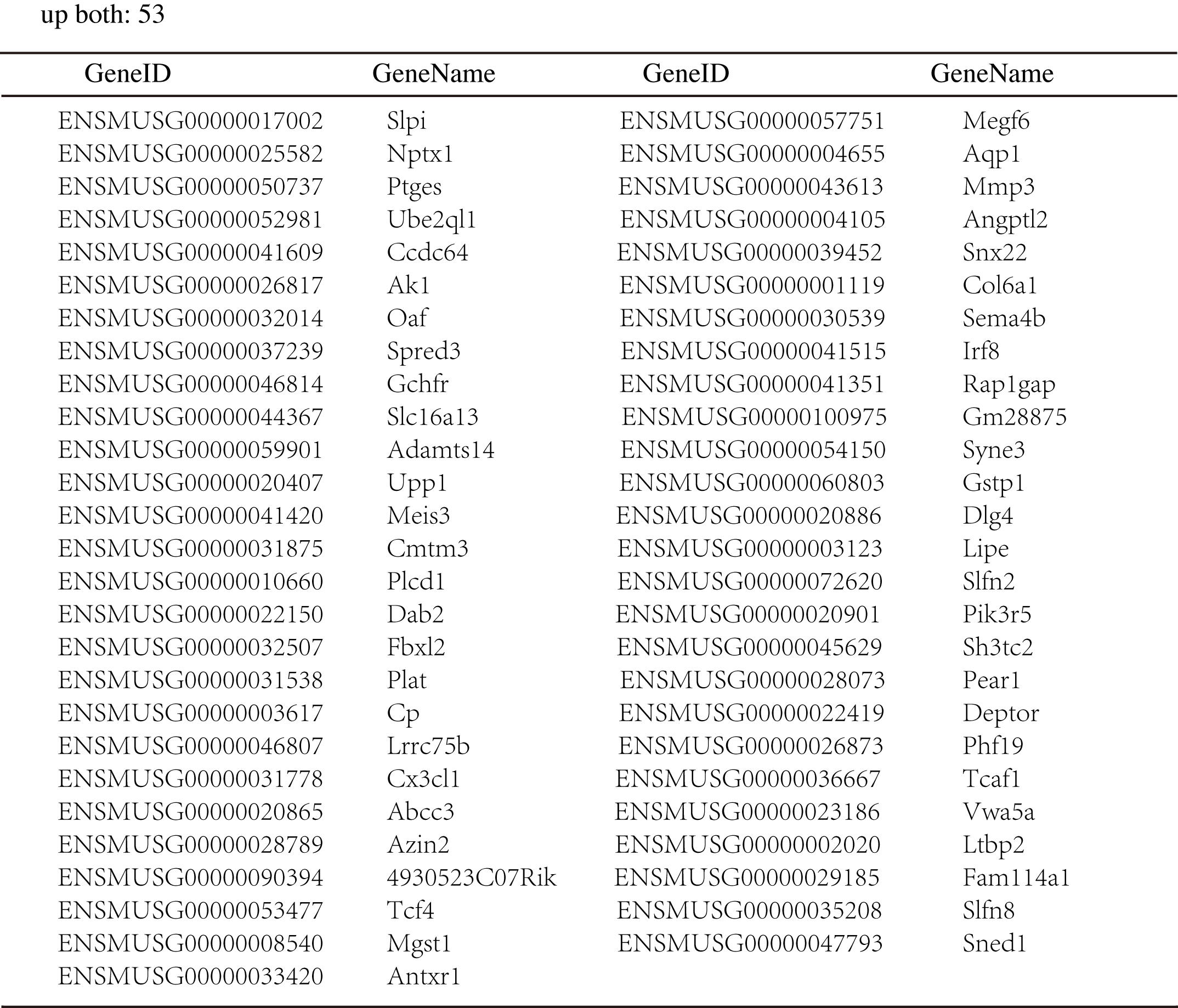
**

**Table s2.** List of 59 genes down-regulated in RH-TS cells as compared to parental cells and RL-TS cells.

**
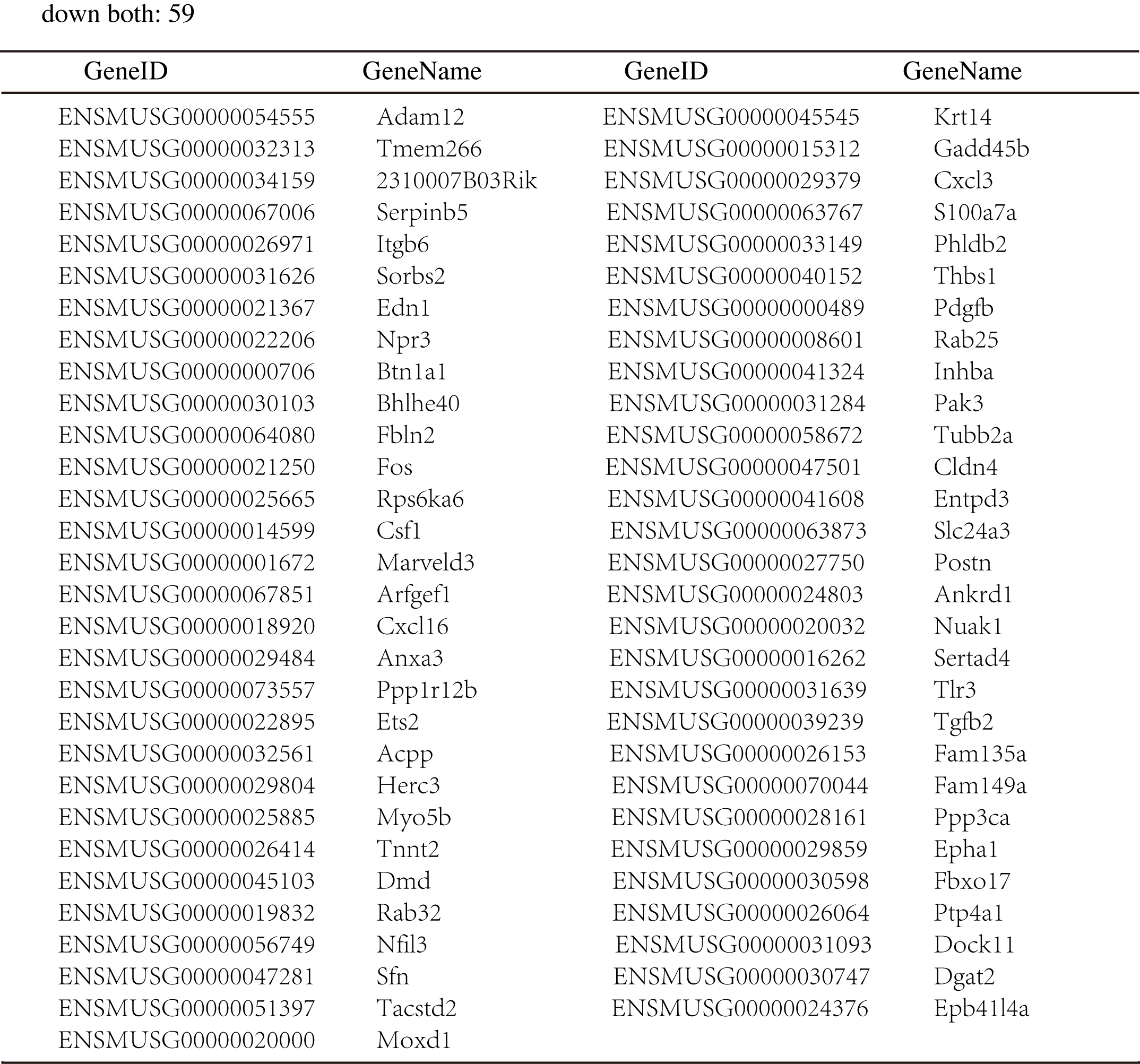
**

**Table s3.** The primers for real-time PCR analysis.

**
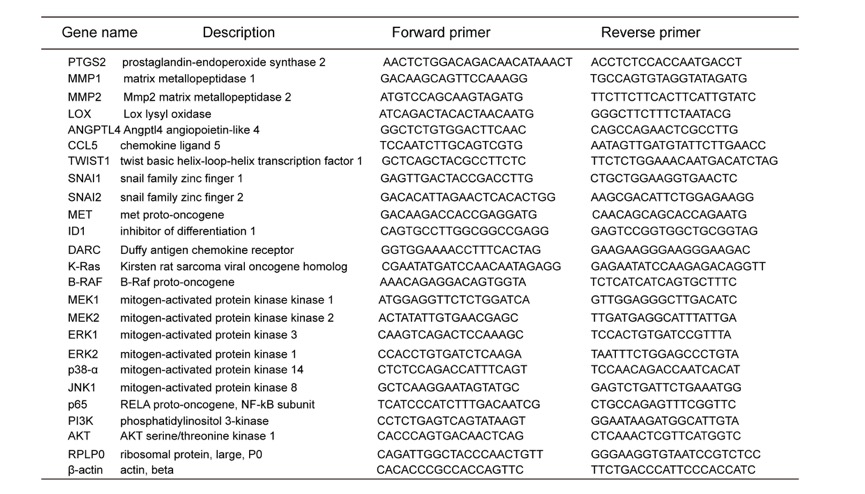
**
